# Highly Robust Sn‐Based MAX Anodes Constructed via Ti_6_C Octahedral Outer Immobilization and A‐Layer Fe Inner Anchoring for Long‐Life Lithium‐Ion Batteries

**DOI:** 10.1002/advs.75085

**Published:** 2026-04-10

**Authors:** Yu‐ang Lei, Yuchen Guo, Linkai He, Yi Tang, Junhui Zou, Yangyang Xie, Xiaodie Xuan, Zhao Bi, Yu Wang, Ting Liu, Yunqing Kang, Taotao Ai, Yusuke Yamauchi, Chenhui Yang

**Affiliations:** ^1^ School of Chemistry and Chemical Engineering Northwestern Polytechnical University Xi'an Shaanxi P. R. China; ^2^ College of Materials Science and Engineering Xi'an University of Science and Technology Xi'an Shaanxi P. R. China; ^3^ State Key Laboratory of Solidification Processing Center For Nano Energy Materials School of Materials Science and Engineering Northwestern Polytechnical University Xi'an Shaanxi P. R. China; ^4^ Department of Materials Process Engineering, Graduate School of Engineering Nagoya University Nagoya Japan; ^5^ School of Materials Science and Engineering Shaanxi University of Technology Hanzhong Shaanxi P. R. China; ^6^ Australian Institute For Bioengineering and Nanotechnology (AIBN) The University of Queensland Brisbane Queensland Australia

**Keywords:** cycling stability, lithium storage, MAX phase, Sn‐based anode

## Abstract

Tin (Sn)‐based anodes offer notable potential for lithium‐ion batteries (LIBs) due to their high theoretical capacity and abundance. However, incomplete lithium insertion and severe volume expansion cause capacity degradation and electrode pulverization, limiting the practical application. To overcome these challenges, we utlize the unique MAX phase structure to stabilize the Sn in the A‐layer between Ti_6_C octahedra while using Fe as inner anchors in the same layer to strongly enhance the Ti–Sn bonding. Based on this design, the highly robust Sn‐based anode materials — layered Ti_2_Sn_1‐_ *_x_* Fe*_x_* C solid‐solution MAX phases with refined grains — are synthesized via a feasible atmosphere sintering followed by ball milling method. The effects of different Fe contents (*x* = 0, 0.10, 0.20, 0.33) on structural stability and lithium storage capacity are also clarified. Among them, the Ti_2_Sn_0.67_Fe_0.33_C electrode exhibits a high specific capacity of 480 mAh g^−1^ at 0.05 A g^−1^ and maintains a specific capacity of 210 mAh g^−1^ after 2000 cycles at 1 A g^−1^ with an average coulombic efficiency of 99.55%, outperforming most of previously reported MAX anode materials. This study presents new material design strategies that help overcome the intrinsic limitations of Sn‐based anodes, while simultaneously expanding the MAX‐phase family.

## Graphical abstract

How can Sn‐based anodes achieve high capacity and long‐life cycling? This work unlocks the potential anode structure by confining Sn in the A layer of MAX phase with Ti_6_C octahedra as outer immobilization and Fe as inner anchors. The introduction of Fe dramatically strengthens the Ti–Sn bond to enhance structural integrity, modulate interfacial kinetics, and promote stable lithium storage.

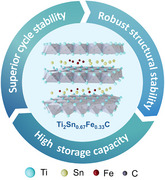

## 1 Introduction

Electrochemical energy storage is critical for grid stabilization and renewable energy integration. Due to high energy density, long cycle life, wide operating temperature range, and low self‐discharge rate [1, 2, 3, 4], lithium‐ion batteries (LIBs) are the most widely used electrochemical energy storage devices for improving energy efficiency and enhancing the stability of power grid operations [5]. Graphite, as the most common anode material in LIBs, offers low cost and good chemical stability, but it also suffers from safety hazards at high current densities, poor cycle performance, and low theoretical capacity (370 mAh g^−1^) [6, 7]. It is worth noting that, due to its alloying reaction with lithium, tin (Sn) has an extremely attractive theoretical capacity as an anode material, reaching 994 mAh g^−1^ (for Li_4.4_Sn), significantly exceeding the capacity of graphite [8, 9]. However, common Sn‐based anodes undergo severe volume expansion (∼250%) during lithiation/delithiation, leading to structural pulverization, rapid capacity fading, low coulombic efficiency, and poor cycling stability [10]. Consequently, to solve this problem, it is crucial to develop a high‐performance Sn‐based anode material that delivers both excellent cycling stability and high capacity.

Common strategies to mitigate these issues involve coating Sn with carbon‐based materials or reducing Sn to the nanoscale, which have proven effective in enhancing cycling stability [11, 12, 13]. Moreover, a unique strategy for stabilizing Sn variations using covalent octahedral atomic layers has been reported. It has been found that the problem of over‐swelling during the reaction of Sn with Li^+^ can be solved by introducing Sn into the A‐site of the MAX, this structure not only releases the stress, but also prevents the Sn particles from swelling and crushing during the lithiation process [14]. MAX phases are hexagonal ternary carbides or nitrides with the general formula M*_n_*_+1_AX*_n_* composed of transition metals (M), group 13–16 elements (A), and carbon/nitrogen (X) [15, 16]. The MAX structure can be regarded as alternating layers of M_6_X octahedra and A‐site atom layers. In the M_6_X octahedral layers, M atoms and X atoms are bonded by strong covalent bonds to form an octahedral structure. The A‐atom layer is sandwiched between the M_6_X layers and located in the octahedral gap position. When Sn atoms at the A‐sites undergo alloying reactions, their volume expansion is restricted by the stable M_6_X layers. Notably, their dense layered structure enables much higher volumetric capacities than graphite, positioning them as highly promising lithium‐ion storage materials, particularly for space‐constrained devices like micro‐sensors and medical implants [17, 18]. For example, Zhao et al. [19] reported the interaction of Nb_2_SnC with lithium ions, demonstrating that the alloying reaction causes the MAX to decompose into smaller particles, and that the reversible capacity of MAX increases with cycles, with an initial capacity of 87 mAh g^−1^ (at 0.5 A g^−1^) of Nb_2_SnC increasing to 150 mAh g^−1^ after 600 charge/discharge cycles. Li et al. [20] reported that V_2_SnC prepared by the molten salt method was investigated as a Li storage anode, exhibiting a high specific capacity of 490 mAh g^−1^, and suggested that the Sn‐Li alloying reaction occurs at the edge of the V_2_SnC phase. Tang et al. [21] achieved uniform in situ‐grown Sn nanoparticles by ball‐milling Ti_2_SnC after high‐temperature heat treatment, and introducing moderate mechanochemical decomposition and defects in the MAX, and this nanoscale composite exhibited excellent electrical conductivity, providing a large number of active sites and shortened ion transport paths, which was recycled for 400 cycles at a current density of 0.1 A g^−1^ exhibiting an excellent long‐term cycling performance of 569 mAh g^−1^. Despite these advantages, MAX materials still face challenges as the A‐layer elements may be partially deintercalated during prolonged cycling, eventually leading to the structural collapse and capacity fading [22]. To suppress such structural degradation caused by the repeated insertion and extraction of lithium ions, introducing other elements into the A‐site has emerged as an effective strategy to enhance the structural stability of MAX materials. For example, Li et al. [23] successfully introduced Fe‐group (Fe, Co, and Ni) elements into the A‐layer of the MAX using an in situ synthesis method, partially replacing Sn atoms in V_2_SnC. Further, experimental and computational studies confirmed that the MAX structure was most stable when the Sn:Fe ratio was 2:1. Hu et al. [24] prepared V_2_(Sn, A)C (A = Ni, Co, Fe) MAX phases, finding that Ni, Sn, and V synergistically enhanced stability and lowered electrochemical reaction barriers. These results suggest that introducing Fe‐group elements into the A‐sites alters the crystal structure and interlayer spacing of the MAX [23, 25, 26].

In this study, we incorporated Fe into the A‐site Sn layers between stable Ti_6_C octahedral layers, which can enhance the structural stability of MAX materials and restrict the volume expansion of Sn during charging and discharging. Ti_2_Sn_1‐_ *_x_* Fe*_x_* C (*x* = 0.10, 0.20, 0.33) MAX phase materials were synthesized, and their performance as LIB anode materials was evaluated. Through comprehensive experiments and calculations, we find that Fe incorporation enhances the binding energy between Ti and Sn of the MAX for structural stability and optimizes Sn utilization in the A‐site. The resulting ball‐milled, A‐site solid‐solution Ti_2_Sn_0.67_Fe_0.33_C exhibits superior Li storage capacity, excellent rate capability, and outstanding cycling stability: a specific capacity of 480 mAh g^−1^ at 0.05 A g^−1^ and 210 mAh g^−1^ at 1 A g^−1^ after 2000 cycles. This study proposes a new material design concept to overcome the inherent limitations of Sn anode materials, while expanding the MAX material system and broadening its application prospects.

## 2 Results and Discussion

### 2.1 Structural Evolution of Ti_2_Sn_1‐_ *_x_* Fe*_x_* C co‐reinforced With Ti_6_C Octahedra and Fe

**FIGURE 1:**
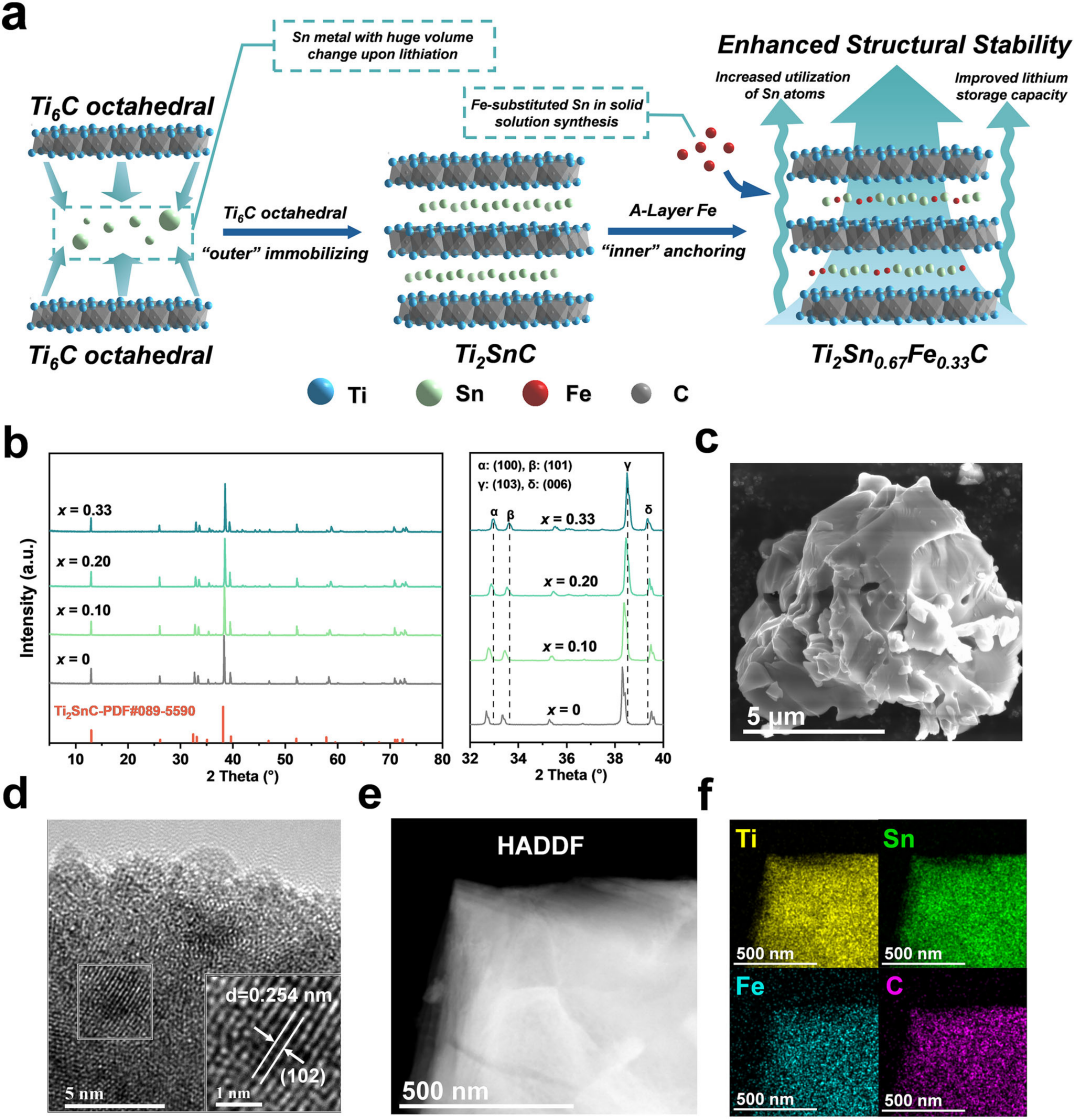
(a) Schematic diagram of Ti_2_Sn_1‐_ *_x_* Fe*_x_* C before and after the introduction of Fe. (b) XRD patterns of the as‐synthesized Ti_2_Sn_1‐_ *_x_* Fe*_x_* C samples. (c) SEM image, (d) HRTEM images, (e) HAADF‐STEM micrograph, and (f) its EDS mapping images of Ti_2_Sn_0.67_Fe_0.33_C sample.

As shown in Figure 1a, addressing the challenge of volume expansion in Sn‐based anodes, this novel stable framework structure, Ti_2_Sn_1‐_ *_x_* Fe*_x_* C MAX phase, reinforces the Sn layer with Ti_6_C octahedral layers while introducing Fe into the Sn site to enhance structural stability.

Specifically, the Ti_2_Sn_1‐_ *_x_* Fe*_x_* C MAX phases (*x* = 0, 0.10, 0.20, and 0.33) were synthesized by atmospheric sintering under Ar using thoroughly ground mixtures of Ti, Sn, Fe, and TiC in designed stoichiometric ratios. To further improve the phase purity of the solid‐solution MAX samples, two sets of precursor combinations (Ti, Sn, Fe, C) and (Ti, Sn, Fe, TiC) were comparatively employed. As shown in Figure S1, X‐ray diffraction (XRD) by using the former precursor set confirms the formation of Ti_3_SnC_2_ impurities, which arises from the Sn–Fe alloy–induced generation of Ti_5_Sn_3_, the known precursor of Ti_3_SnC_2_ [27, 28, 29]. Even when the sintering temperature decreases to 1050°C, the impurity Ti_3_SnC_2_ phase is still present. Moreover, when the latter uses a mixture of Ti, Sn, Fe, and TiC as raw materials, the decreased proportion of Ti inhibits the formation of Ti_5_Sn_3_ and Ti_3_SnC_2._ To prove this point, as shown in Figure S2, a sample of *x* = 0.33 was sintered at 800°C, and it could be found that the main components are TiC, Ti_6_Sn_5_, and FeSnTi, and Ti_5_Sn_3_ was not generated. Therefore, we selected the latter raw material to synthesize MAX with different Ti_2_Sn_1‐_ *_x_* Fe*_x_* C contents. We found that Ti_2_SnC prepared using TiC as raw material exhibits significantly higher purity than that reported in the literature, containing virtually no TiC or Ti*_x_* Sn*_y_* impurities [30, 31].

The XRD patterns of Ti_2_Sn_1‐_ *_x_* Fe*_x_* C after acid washing and pulverization treatment is shown in Figure 1b, and the diffraction peaks of the sample can correspond well to the M_2_AX phase in the whole composition range [32, 33]. This clearly demonstrates the successful preparation of the solid solution. In addition, it can be noticed that the (100), (101), and (103) peaks of the solid‐solution Ti_2_Sn_1‐_ *_x_* Fe*_x_* C shift to higher angles as the Fe content increases and the Sn content decreases, and the distance between the (006) and (103) peaks becomes smaller, a phenomenon that also occurs in other similar studies [34, 35, 36, 37]. As shown in Figure S3, when the Fe ratio is further increased to *x* = 0.50, the main phase becomes TiC, and a large amount of Fe_2_Ti impurities are still present, indicating that the Fe element doesn't completely enter the A‐layer. Continued addition of Fe leads to a decrease in the MAX content and an increase in impurities, making it impossible to synthesize high‐purity products. In other words, it can be inferred that Fe only occupies one‐third of the A‐site. Therefore, we only studied different compositions (*x* = 0, 0.10, 0.20, and 0.33) of Ti_2_Sn_1‐_ *_x_* Fe*_x_* C.

Figure S4 shows the XRD comparison of the Ti_2_Sn_1‐_ *_x_* Fe*_x_* C phases before and after ball milling. It can be found that the the full width at half maximum increases and weaker after ball milling, which is due to amorphization and the reduction of the particle sizes from Scherrer's equation [38]. The enhancement of the (002) peak may be due to one of the following: The ductility of the metal bond between the M and the A‐layer allows interlayer movement without destroying the crystal structure, meaning that the (002) lattice plane of the c‐axis remains intact. Consequently, the MAX material can exfoliate and slip preferentially along the interlayer direction during ball milling to form more layered structures [39].

In order to further determine the microscopic morphology of Ti_2_Sn_1‐_ *_x_* Fe*_x_* C grains, scanning electron microscopy (SEM) was used. As shown in Figure 1c and Figure S5, It is observed that there is a clear multilayer structure at the boundaries, which exhibits a distinct MAX appearance [40]. Figure S6 demonstrates the SEM image of Ti_2_Sn_0.67_Fe_0.33_C grains after ball milling, indicating that the particle size reduces after ball milling, which further validates the results of the XRD analysis. The proportion of elements in the Ti_2_Sn_1‐_ *_x_* Fe*_x_* C samples was quantitatively analyzed by energy dispersive spectroscopy (EDS). As shown in Table S1, the quantitative analysis confirms that the actual percentage of Fe in the A‐site is very close to the designed compositions (*x* = 0.10, 0.20, and 0.33), with the values of 0.10, 0.22, and 0.31, respectively.

As displayed in Figure 1d and Figure S7, the high‐resolution transmission electron microscopy (HRTEM) image of Ti_2_Sn_0.67_Fe_0.33_C shows that the lattice streak with a size of 0.254 nm represents the (102) peak of the crystal, indicating the successful preparation of the solid‐solution MAX phase. The high‐angle annular dark‐field scanning transmission electron microscopy (HAADF‐STEM) image of Ti_2_Sn_0.67_Fe_0.33_C in Figure 1e reveals that there is a clear multilayer structure at the edge, which is consistent with the SEM result and the reported Sn‐based MAX [14, 19, 20]. As shown in the EDS mapping of TEM (Figure 1f), the four elements of Ti, Sn, Fe, C are uniformly distributed in the Ti_2_Sn_0.67_Fe_0.33_C grain, which is further evidence of good elemental dispersion in the target sample.

**FIGURE 2:**
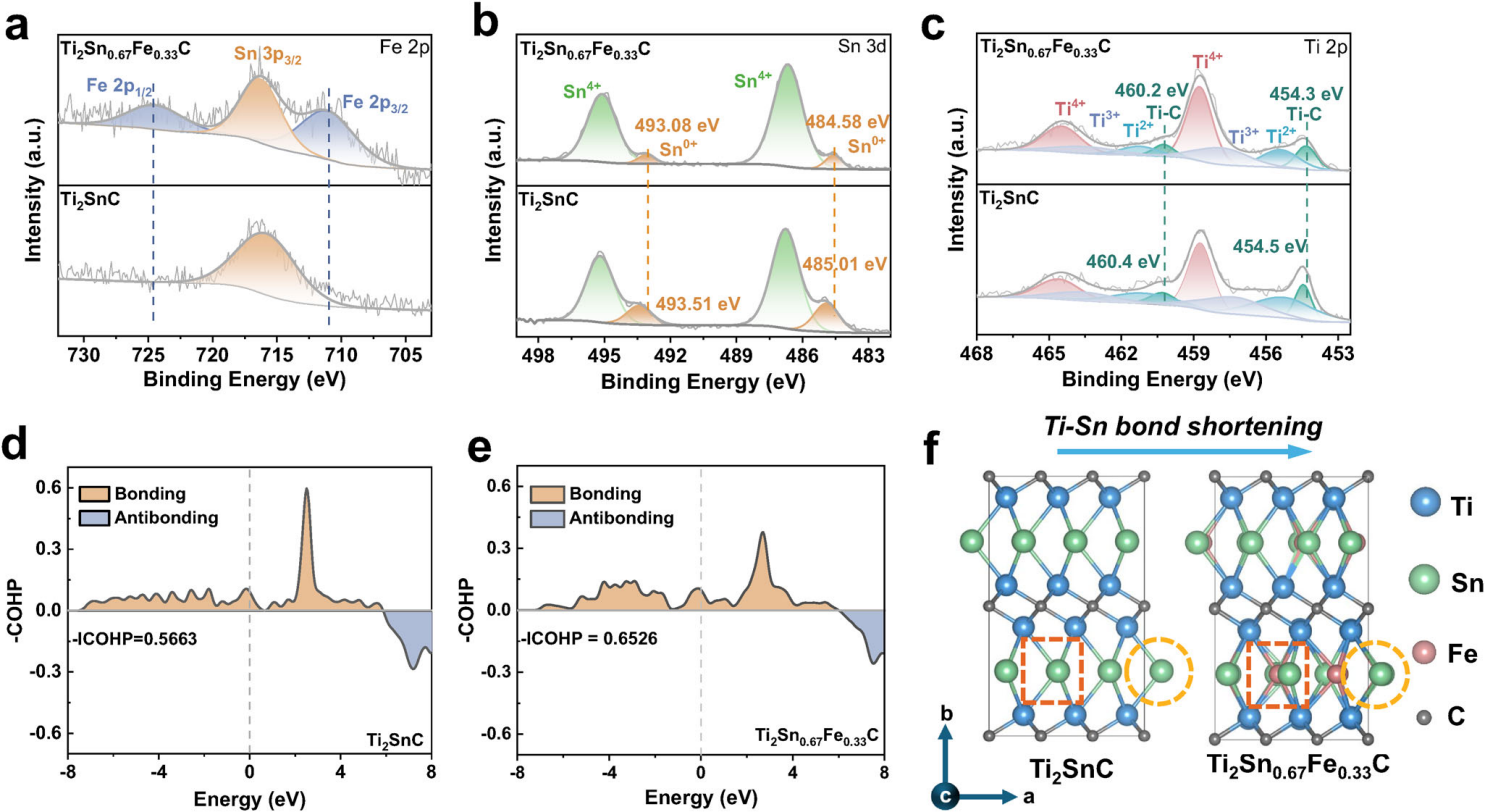
(a) Fe 2*p*, (b) Sn 3*d*, (c) Ti 2*p* region of XPS spectra of Ti_2_Sn_0.67_Fe_0.33_C and Ti_2_SnC samples. Crystal orbital Hamiltonian population (COHP) analysis of Ti‐Sn binding energy changes of (d) before and (e) after (*x* = 0.33) Fe introduction. (f) Schematic diagrams of the crystal structures before and after (*x* = 0.33) Fe introduction.

The valence states of surface elements and electronic properties of Ti_2_Sn_0.67_Fe_0.33_C and Ti_2_SnC can be explored by X‐ray photoelectron spectroscopy (XPS). Figure S8 indicates the total XPS spectra of Ti_2_Sn_0.67_Fe_0.33_C and Ti_2_SnC samples, in which signals of Sn 3*d*, Ti 2*p*, Fe 2*p*, C 1*s*, and O 1*s* are observed. Figure 2a–c demonstrate the XPS spectra of Fe 2*p*, Sn 3*d*, and Ti 2*p* of Ti_2_Sn_0.67_Fe_0.33_C and Ti_2_SnC.

The narrow scanning spectra of Fe 2*p*_3/2_ (711.04 eV) and Fe 2*p*_1/2_ (724.15 eV) can be observed [25, 26], whereas no Fe 2*p* signals appear in Ti_2_SnC. For Sn, the peaks corresponding to metallic Sn in Ti_2_Sn_0.67_Fe_0.33_C (Sn^0^ at 484.58 and 493.08 eV) and Sn oxide produced by surface oxidation can be observed [21, 41]. It can be observed that the introduction of Fe causes the positions of the Sn 3d orbital peaks to shift toward lower energies (from 485.01 to 484.58 eV, and from 493.51 to 493.08 eV). Since the electronegativity of Sn atoms is higher than that of Fe atoms, when the Sn atoms are replaced by Fe atoms, it decreases the electronic binding energy of Sn 3*d*, resulting in the peaks shift to lower binding energies, and the chemical shift is the result of the change of chemical environment. The Ti 2*p* XPS spectrum reveals that the presence of Ti^4+^ arises from partial oxidation of the MAX surface during ball milling, while the Ti^2+^ and Ti^3+^ species originate from the unoxidized MAX phase bulk. Compared with Ti_2_SnC (454.5, 460.4 eV), the introduction of Fe also causes a slight decrease in the Ti–C bond energies in Ti_2_Sn_0.67_Fe_0.33_C (454.3, 460.2 eV). Through the above analysis, it demonstrates that Fe has been successfully incorporated into the crystal structure of the solid solution.

To study the effect of Fe introduction on the structural stability of the MAX, the change of Ti‐Sn bonding energy before and after Fe introduction was analyzed using the crystal orbital Hamiltonian population (COHP) through first‐principles theoretical calculations. As shown in Figure 2d,e, it can be found that, the trend of Ti‐Sn bonding in bonding orbitals increases after Fe introduction, and the value of ‐ICOHP increases from 0.5663 to 0.6526, which implies that the bond strength between Ti‐Sn is enhanced, and the structure of the overall MAX is more stable, which is consistent with the preceding XPS analysis. Figure 2f shows the calculated MAX‐phase crystal structures before and after Fe incorporation. It can be observed that after introducing Fe, some Ti‐Sn bond lengths become shorter, indicating strengthened bond energies.

Figure S9 shows the hysteresis lines of Ti_2_Sn_1‐_ *_x_* Fe*_x_* C with different Fe contents at 300 K. It is observed that the hysteresis loops follow the “S‐shape” characteristic, and the Fe element in Ti_2_Sn_1‐_ *_x_* Fe*_x_* C shows paramagnetic behavior, which is in agreement with other research reports [23]. The highest saturation paramagnetic strengths of the Ti_2_Sn_1‐_ *_x_* Fe*_x_* C are 0.159 emu g^−1^ (Ti_2_Sn_0.9_Fe_0.1_C), 0.213 emu g^−1^ (Ti_2_Sn_0.8_Fe_0.2_C), and 0.235 emu g^−1^ (Ti_2_Sn_0.67_Fe_0.33_C), respectively, and the saturation paramagnetic strengths increase with the increase of the contents of Fe elements.

### 2.2 Long‐life Li‐ion Storage Behaviors of Highly Robust Ti_2_Sn_0.67_Fe_0.33_C Anodes

**FIGURE 3:**
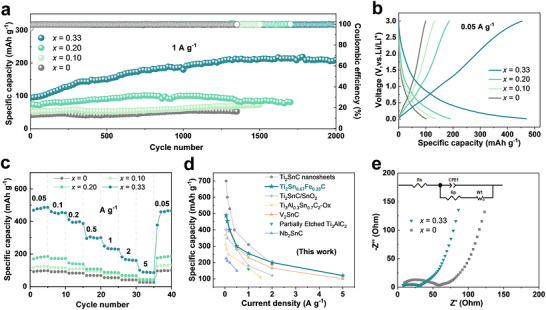
Li | 1 m LiPF_6_ in EC: DMC (1:1) | Ti_2_Sn_1‐_ *_x_* Fe*_x_* C MAX phases: (a) Long‐term cycling performance of four Ti_2_Sn_1‐_ *_x_* Fe*_x_* C electrodes at 1 A g^−1^. (b) Charge/discharge profiles of four Ti_2_Sn_1‐_ *_x_* Fe*_x_* C electrodes from 0.01 to 3 V. (c) The rate performance of four Ti_2_Sn_1‐_ *_x_* Fe*_x_* C electrodes after long cycles at different current densities. (d) Comparison of the MAX and the derivatives as anodes in LIBs. (e) Nyquist plots of Ti_2_Sn_0.67_Fe_0.33_C and Ti_2_SnC electrodes.

Although previous studies suggested that an Sn:Fe ratio of 2:1 corresponds to the highest structural stability of Ti_2_Sn_1‐_ *_x_* Fe*_x_* C, electrochemical performance depends on additional factors such as Li^+^ transport kinetics and the activation of Sn species. Therefore, multiple Ti_2_Sn_1‐_ *_x_* Fe*_x_* C compositions were systematically evaluated to experimentally clarify the relationship between structural stability and lithium‐storage behaviors. The electrochemical performance of the Ti_2_Sn_1‐_ *_x_* Fe*_x_* C powders as a LIB anode was investigated using two‐electrode Li‐ion half‐cell testing. Conductive carbon black and polyvinylidene fluoride were added to Ti_2_Sn_1‐_ *_x_* Fe*_x_* C at a ratio of 10 wt.% each to prepare the working electrodes, with Li metal foil used as the counter electrode. Figure 3a demonstrates the cycling performance of four Ti_2_Sn_1‐_ *_x_* Fe*_x_* C electrodes at 1 A g^−1^, showing that the specific capacity increases with cycling. This is because, as the cycle increases, the A‐layer undergoes partial etching, causing Sn atoms to accumulate at the edges of the MAX grains, Sn atoms located deeper in the A‐layer become electrochemically accessible. Table S2 shows the cycling performance and average coulombic efficiency of Ti_2_Sn_1‐_ *_x_* Fe*_x_* C. Compared to the capacity of pure Ti_2_SnC (50 mAh g^−1^), the specific capacity of the Ti_2_Sn_0.67_Fe_0.33_C is higher than 210 mAh g^−1^ under longer cycling. Since Fe exhibits negligible alloying reaction with Li^+^, this facilitates easier utilization of Sn located deep within the A‐layer of the MAX. Moreover, the phase transition energy barrier of the Ti_2_Sn_1‐_ *_x_* Fe*_x_* C phase is significantly lower than that of intermetallic compounds [42, 43], thereby exhibiting superior reversibility in lithium alloying/dealloying and enhanced cycling stability. Specifically, the experimental results demonstrate that the specific capacity and cycling stability improve progressively with the increase of Fe content. When *x* = 0.33, the sample exhibits optimal electrochemical performance, providing strong experimental evidence, confirming the rationality of the composition design.

The specific capacity of the Ti_2_Sn_0.67_Fe_0.33_C electrode at a current density of 1 A g^−1^ increased from 95 to 210 mAh g^−1^ after 2000 cycles, with an average coulombic efficiency of 99.55%. This demonstrates that introducing Fe into the A‐site significantly enhances its cycling performance. The introduction of Fe enhances the bonding between Ti and Sn, thereby stabilizing the overall structure of the MAX and enabling Li^+^ to alloy with Sn atoms located deep within the A‐layer. The overall framework remains partially preserved despite local structural evolution during cycling, a finding that can be verified through the preceding calculations. In short, the introduction of Fe enhances the crystalline structural stability of the solid solution.

Figure 3b demonstrates the galvanostatic charge–discharge (GCD) curves of four Ti_2_Sn_1‐_ *_x_* Fe*_x_* C electrodes after long cycling. It can be seen that there is no obvious electrochemical plateau. This is consistent with the shape of the alloying reaction curve and suggests that the A‐site metal of the MAX undergoes an alloying reaction with Li ions. Figure 3c shows the rate performance of four Ti_2_Sn_1‐_ *_x_* Fe*_x_* C after long cycling tested at different current densities from 0.05 to 5 A g^−1^. The Ti_2_Sn_0.67_Fe_0.33_C electrode delivers a maximum reversible capacity of 480 mAh g^−1^ at 0.05 A g^−1^. Compared to the rate performance of the Ti_2_Sn_1‐_ *_x_* Fe*_x_* C without cycling in Figure S10, both the capacity and rate properties are greatly improved after long cycling. The specific capacity of the Ti_2_Sn_0.67_Fe_0.33_C electrode increases from 170 to 480 mAh g^−1^ at 0.05 A g^−1^, which is attributed to the fact that the A‐site is electrochemically partially etched after repeated cycling and the Sn atoms are stacked at the edge of the MAX in order to provide more reaction sites. Furthermore, compared to other MAX that are not nanosheets [14, 18, 19, 20, 22, 36], the Ti_2_Sn_1‐_ *_x_* Fe*_x_* C in this study exhibits a higher specific capacity, as shown in Figure 3d.

The kinetic properties of the working electrode were explored by electrochemical impedance spectroscopy (EIS) measurements. Figure 3e and Figure S11 illustrate the Nyquist plots of the initial state of the Ti_2_Sn_1‐_ *_x_* Fe*_x_* C before cycling with different Fe ratios. The Ti_2_Sn_0.67_Fe_0.33_C electrode has a smaller charge transfer resistance (*R*_ct_, 25.46 Ω) than those of the Ti_2_Sn_0.8_Fe_0.2_C (31.92 Ω), Ti_2_Sn_0.9_Fe_0.1_C (46.95 Ω), and Ti_2_SnC (55.92 Ω), suggesting that, beyond providing structural stabilization, the introduction of Fe also significantly affects the local electronic structure and solid electrolyte interface (SEI) formation. Theoretically, the 3*d* orbital characteristics of iron contribute to optimizing electron transfer, thereby reducing the resistance of electrochemical reactions [44]. Furthermore, by acting as a rigid “inner anchor” within the A‐layer, Fe effectively mitigates the local strain induced by the alloying/dealloying of Sn. This structural buffering effect is beneficial for stabilizing the SEI during long‐term cycling, thereby contributing to the improved electrochemical stability and interfacial kinetics [45].

Figure S12 demonstrates the cyclic voltammetry (CV) graphs between 0.01 and 3 V (vs. Li/Li^+^) of different proportions of solid solutions at a sweep rate of 0.1 mV s^−1^. It can be found that no new redox peaks were observed, but the area of the CV curves increased. The increase in peak area indicates that the lithium storage capacity of the solid solution increases with rising Fe content, consistent with the results obtained from the GCD curve. The CV curves of the Ti_2_Sn_0.67_Fe_0.33_C electrode in the first two cycles at a sweep rate of 0.1 mV s^−1^ are illustrated in Figure S13. And there is a broad irreversible reduction peak at 0.75 V, which can be explained by the formation of a SEI‐layer [46, 47]. The reduction peak in CV curves at 0.18–0.59 V corresponds to the Li_4.4_Sn alloy formation process driven by lithiation of Sn (Sn + 4.4Li^+^ + 4.4e^−^ → Li_4.4_Sn), while the oxidation peak at about 0.60 V characterizes the Li_4.4_Sn alloy delithiation reaction (Li_4.4_Sn → Sn + 4.4Li^+^ + 4.4e^−^), suggesting the electrochemical reversibility of the Li‐Sn alloying/de‐alloying reaction in the tin‐based anode [14, 48]. It is worth noting that the significant oxidation peak at 0.1 V in the second CV curve suggests that there is an oxidative dissolution behavior of trace deposited Li at the interface.

### 2.3 Long‐term Li‐ion Storage Mechanisms of Highly Stable Ti_2_Sn_0.67_Fe_0.33_C Anode

**FIGURE 4:**
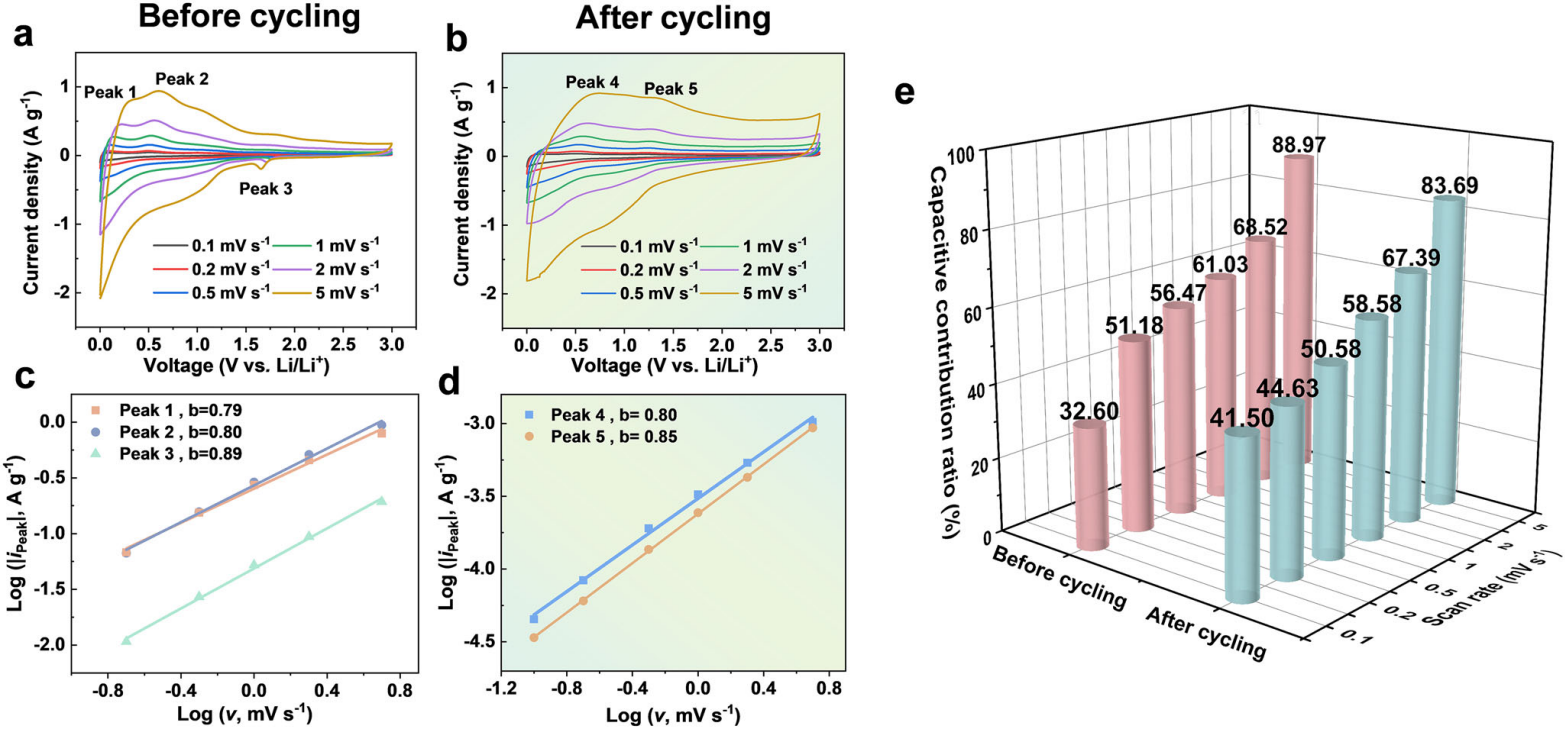
CV curves of the Ti_2_Sn_0.67_Fe_0.33_C electrode (a) before and (b) after 2000 cycles at different scan rates. The plot of the relationship between peak current response and scan rate of the Ti_2_Sn_0.67_Fe_0.33_C electrode (c) before and (d) after cycling. (e) Capacitance contribution ratio of the Ti_2_Sn_0.67_Fe_0.33_C electrode before and after cycling.

In order to further elucidate the long‐term Li‐ion storage mechanism of the stable Ti_2_Sn_0.67_Fe_0.33_C electrode before and after 2000 cycles, CV tests at scan rates from 0.1 to 5.0 mV s^−1^ are shown in Figure 4a,b, where the peak currents are increased with the increase of scanning rates. Compared to the uncycled Ti_2_Sn_0.67_Fe_0.33_C electrode, the cycled Ti_2_Sn_0.67_Fe_0.33_C exhibits the increased integral area at scan rates of 0.1–5.0 mV s^−1^, particularly displaying markedly broader contours in the 1.5–3.0 V region. This enhancement originates from the etched MXene portion at the MAX edge [49]. The redox peaks (Peak 1 and 2) formed by the Sn alloying reaction remain present even after prolonged cycling. Peak 3 is the reduction peak of the outer layer of trace oxides formed by the exudation of some of the Sn metal during the ball milling process [21]. After cycling, the disappearance of Peak 3 stems from the expansion/deactivation of oxides following repeated cycling.

The linear fit of the peak current (|*i*_peak_|) in Figure 4a,b is shown in Figure 4c,d. Using Equations S1 and S2 to calculate the *b‐*values of Peaks 1 and 2 gives values of 0.79 and 0.80, respectively. These values are intermediate between capacitance‐ and diffusion‐controlled processes and are consistent with the reported Sn alloying reaction [50]. After cycling, the increased *b*‐values of 0.80 for Peak 4 and 0.85 for Peak 5, demonstrate the pronounced capacitive characteristics of the alloying reaction. The *b‐*value of peak 3 is 0.89, which is primarily capacitance‐controlled. It has previously been reported that the kinetic behavior of Sn oxide exhibits a high *b*‐value characteristic (0.85–0.92) when the oxide is a very thin flake [51].To further quantify the contribution of capacitance to the overall current response, we employed the method of Wang et al. [52] to quantitatively measure the capacitive effect contribution at different scan rates. Calculations using Equation S3 reveal that at 1 mV s^−1^, the fitted capacitive contributions for the Ti_2_Sn_0.67_Fe_0.33_C electrode and the Ti_2_Sn_0.67_Fe_0.33_C electrode after extended cycling were 61.0% and 58.6%, respectively (Figure S14). As shown in Figure 4e, the surface capacitance contribution was calculated using Equation S3. The capacitance contributions of the Ti_2_Sn_0.67_Fe_0.33_C electrode after cycling from 0.1 to 5 mV s^−1^ are calculated to be 41.50%, 44.63%, 50.58%, 58.58%, 67.39%, and 83.69%, which are lower than those of the Ti_2_Sn_0.67_Fe_0.33_C anode at most scan rates before cycling (32.60%, 51.18%, 56.47%, 61.03%, 68.52%, 88.97%). This suggests that in cycling, electrochemical behaviors shift toward diffusion control, and Sn agglomeration at A‐sites and migration to phase edges enables greater participation of Sn in alloying reactions. To verify this interpretation, the dQ/dV curves of the Ti_2_Sn_0.67_Fe_0.33_C electrode at different cycles were obtained at a current density of 1 A g^−1^. As shown in Figure S15, with increasing cycle number, the alloying reaction peaks of Sn gradually intensify and reach a maximum after 1000 cycles. Beyond 1000 cycles, the further increase in lithium‐storage capacity mainly arises from the enlarged curve area in the voltage range of 1.5–3.0 V, which is attributed to the contribution of MXene generated by edge etching of the MAX phase during cycling.

**FIGURE 5:**
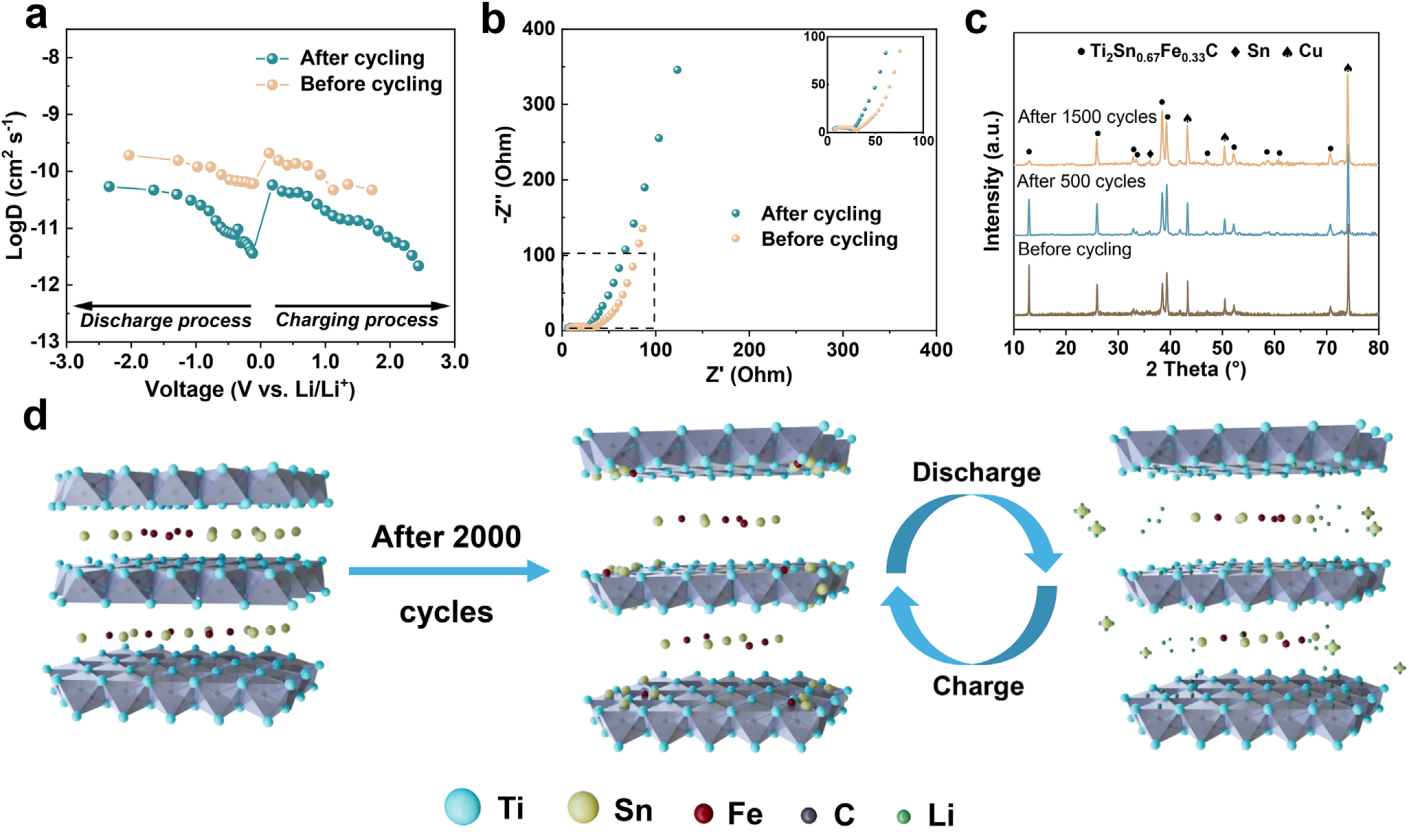
The Ti_2_Sn_0.67_Fe_0.33_C electrode before and after 2000 cycles: (a) Ion diffusion values calculated from GITT, (b) Nyquist plots. (c) XRD patterns of the Ti_2_Sn_0.67_Fe_0.33_C under different cycles. (d) Li‐ion storage mechanism diagram of the Ti_2_Sn_0.67_Fe_0.33_C electrode.

Galvanostatic Intermittent Titration Technique (GITT) was carried out to measure the change of the diffusion coefficient (Log D) of the Ti_2_Sn_0.67_Fe_0.33_C electrode before and after cycling. As shown in Figure 5a, compared with the Ti_2_Sn_0.67_Fe_0.33_C electrode before cycling, the diffusion coefficient after cycling becomes smaller, indicating slower Li^+^ diffusion kinetics. This decrease is mainly attributed to the structural and interfacial changes that occur during prolonged cycling. Initially, Li^+^ can directly reach the active Sn species that are exposed on the surface or at defect sites, enabling relatively fast alloying reactions. After long‐term cycling, repeated alloying/dealloying induces pulverization of Sn, partial detachment of the A‐layer, and the formation of a thicker Sn/Li*_x_* Sn‐rich layer as well as interfacial by‐products. These changes increase the tortuosity of Li^+^ diffusion pathways, thereby hindering the transport of Li^+^ and resulting in a lower diffusion coefficient.

Figure 5b shows the EIS results of the Ti_2_Sn_0.67_Fe_0.33_C electrode before and after cycling, and it can be found that the semicircle diameter decreases with increasing cycles, possibly due to decreased charge transfer resistance. This indicates that it undergoes a self‐activation process, resulting in rapid electron transfer during the electrochemical reaction, which may be one reason for the increased capacity and is a characteristic of MAX‐phase electrode materials [21, 47].

To investigate the changes in the crystal structure of MAX materials during electrochemical reactions, *ex situ* XRD and electrochemical charge tests were carried out on the Ti_2_Sn_0.67_Fe_0.33_C at different voltages, as shown in Figure S16. In the charging process, the (002) peak first shifts to a lower angle before shifting to a higher angle, due to the change of crystalline interlayer spacing. During the initial charging stage (0–1 V), the Sn atoms at the edge of the A‐site expand due to the alloying reaction, resulting in a widening of the interlayer spacing, and the (002) peak shifts to a lower angle (a lower angle). As the further charging process proceeds (1–3 V), the Sn atoms in the depth of the A‐site layer dislodge and Sn vacancies appear [21], and the neighboring M‐site layers then lose support, and the interlayer attraction strengthens, and the spacing between the layers decreases, and the (002) peak shifts to the right. In addition to the change of the (002) peak, the appearance of the diffraction peaks of the Li*_x_* Sn alloy between 17.5 and 30.0 degrees (marked by black club) and the diffraction peaks of Sn metal at 36.1 degrees (marked by black square) during charging indicates the occurrence of the Sn alloying reaction at the A‐site. Finally, in order to investigate the changes of the Ti_2_Sn_0.67_Fe_0.33_C crystal structures before and after cycling, XRD tests of the Ti_2_Sn_0.67_Fe_0.33_C electrodes before cycling, after 500 cycles, and 1500 cycles were carried out, as shown in Figure 5c. As cycling increases, the diffraction peaks of Sn slightly increase in intensity, while the (002) peak of Ti_2_Sn_0.67_Fe_0.33_C almost disappears. This is because, after repeated electrochemical cycling, the (002) diffraction peak of the Ti_2_Sn_0.67_Fe_0.33_C gradually disappeared. This can be attributed to the repeated lithiation/delithiation‐induced structural change, the partial conversion of Sn into Li*_x_* Sn alloys, and the formation of amorphous or oxide phases during cycling. These processes destroy the long‐range order along the c‐axis, leading to the disappearance of the (002) reflection, the same phenomenon can be observed in studies of other MAX [21, 47]. The other diffraction peaks of Ti_2_Sn_0.67_Fe_0.33_C after cycling are still clearly visible, indicating that much of the crystal structure remains intact. This shows that the Sn alloying reaction at the A‐site of the MAX is highly reversible.

As shown in Figure 5d, during lithiation, the MAX undergoes an initial conversion reaction in which Li^+^ ions react with Sn atoms to form Li*_x_* Sn alloys, and the MAX partially decomposes into MXene and metallic Sn. In subsequent charge–discharge cycles, the electrochemical process is dominated by the reversible alloying/dealloying reaction between Sn and Li. The Ti–C framework provides electronic conductivity and structural integrity, buffering the volume changes of the Sn/Li*_x_* Sn phases and enhancing cycling stability. The disappearance of the (002) diffraction peak in the XRD patterns after cycling confirms the partial collapse of the layered structure during this conversion. The introduction of Fe effectively reinforces the structural stability of the MAX, yielding improved cycling performance. After repeated cycling, metallic Sn accumulates at the edges outside the A‐layer of the MAX, which renders Sn located deeper within the A‐layer accessible and thus exposes more active sites. At the same time, the edge regions of the MAX are partially etched into MXene, enabling the MAX to achieve higher lithium storage capacity.

## 3 Conclusion

In summary, we have successfully synthesized highly robust solid‐solution Ti_2_Sn_1‐_ *_x_* Fe*_x_* C MAX for the first time via pressureless sintering. Benefiting from the inherent layered structure of MAX compounds, the Ti_6_C octahedra provide a rigid framework immobilizing A‐site atoms, while Fe anchors within the A‐layer to stabilize the interlayer space. Adjusting the content of Fe in the A‐site atomic layers can control the paramagnetic strength of MAX materials. Among four Ti_2_Sn_1‐_ *_x_* Fe*_x_* C MAX phases, Ti_2_Sn_0.67_Fe_0.33_C exhibits the highest structural stability, best cycling stability, and maximum lithium storage capacity. During the electrochemical cycling of the Ti_2_Sn_0.67_Fe_0.33_C electrode, an increasing number of Sn atoms from the A‐layer participate in the alloying reaction as cycling progresses, reaching a maximum after 1000 cycles. Thereafter, the enhancement of lithium storage capacity is primarily attributed to the generation of MXene through the etching of MAX phase edges. Specifically, after 2000 charge‐discharge cycles, the Ti_2_Sn_0.67_Fe_0.33_C electrodeprovides a specific capacity of 210 mAh g^−1^ at a current density of 1 A g^−1^ with an average coulombic efficiency of 99.55%, and a higher specific capacity of 480 mAh g^−1^ at a current density of 0.05 A g^−1^. For Li‐ion battery anodes, both experimental results and theoretical calculations demonstrate that Fe incorporation optimizes the local chemical environment of Sn, enhances the stability of Sn anchored within the Ti_6_C confined A‐layer, promotes reversible Li^+^ insertion/extraction, and effectively mitigates volume expansion. This prevents structural powdering and enhances the utilization rate of Sn at the A‐site. Our performance surpasses most reported MAX‐phase‐based anode materials, demonstrating that the A‐site solid solution modification strategy offers significant advantages in enhancing the comprehensive performance of electrode materials. This study offers a promising approach for the future development of high‐capacity and long‐life alloy‐based anode materials.

## Conflicts of Interest

The authors declare no competing financial interest.

## Supporting information




**Supporting File**: advs75085‐sup‐0001‐SuppMat.docx.

## References

[1] K. Yang , F. Zhao , C. Li , et al., “Developing a One‐Pot Strategy to Synthesize Metal–Covalent Organic Frameworks as Catalysts for Polysulfide Conversion and Ion Calibrators for Lithium Deposition,” Advanced Functional Materials 35 (2025): 2501980, 10.1002/adfm.202501980.

[2] H. Ji , J. Xiang , Y. Li , et al., “Liquid–liquid Interfacial Tension Stabilized Li‐metal Batteries,” Nature 643 (2025): 1255–1262, 10.1038/s41586-025-09293-4. 40670799

[3] J. Wagner‐Henke , D. Kuai , M. Gerasimov , F. Röder , P. B. Balbuena , and U. Krewer , “Knowledge‐driven Design of Solid‐electrolyte Interphases on Lithium Metal via Multiscale Modelling,” Nature Communications 14 (2023): 6823, 10.1038/s41467-023-42212-7. PMC10603056 37884517

[4] T. Wang , B. Chen , Y. Liu , et al., “Fatigue of Li Metal Anode in Solid‐state Batteries,” Science 388 (2025): 311–316, 10.1126/science.adq6807. 40245125

[5] T. Chen , Y. Jin , H. Lv , et al., “Applications of Lithium‐Ion Batteries in Grid‐Scale Energy Storage Systems,” Transactions of Tianjin University 26 (2020): 208–217, 10.1007/s12209-020-00236-w.

[6] Y. Zhao , Y. Fu , Y. Meng , Z. Wang , J. Liu , and X. Gong , “Challenges and Strategies of Lithium‐ion Mass Transfer in Natural Graphite Anode,” Chemical Engineering Journal 480 (2024): 148047, 10.1016/j.cej.2023.148047.

[7] Y. Dong , C. Liu , M. Rui , et al., “Review on Graphite Anodes for Fast‐Charging Lithium‐Ion Batteries: Mechanism, Modification and Characterizations,” Advanced Functional Materials 35 (2025): 2506190, 10.1002/adfm.202506190.

[8] H. Ying and W.‐Q. Han , “Metallic Sn‐based Anode Materials: Application in High‐Performance Lithium‐ion and Sodium‐ion Batteries,” Advanced Science 4 (2017): 1700298, 10.1002/advs.201700298. 29201624 PMC5700643

[9] H. Zhang , D. Xu , F. Yang , et al., “A High‐capacity Sn Metal Anode for Aqueous Acidic Batteries,” Joule 7 (2023): 971–985, 10.1016/j.joule.2023.04.011.

[10] F. Xin and M. S. Whittingham , “Challenges and Development of Tin‐Based Anode With High Volumetric Capacity for Li‐Ion Batteries,” Electrochemical Energy Reviews 3 (2020): 643–655, 10.1007/s41918-020-00082-3.

[11] X. Zhu , J. Xiao , Y. Chen , et al., “A High‐performance nano‐Sn/G@C Composite Anode Prepared by Waste Carbon Residue from Spent Lithium‐ion Batteries,” Chemical Engineering Journal 450 (2022): 138113, 10.1016/j.cej.2022.138113.

[12] S. Abouali , M. Akbari Garakani , and J.‐K. Kim , “Ultrafine SnO_2_ Nanoparticles Encapsulated in Ordered Mesoporous Carbon Framework for Li‐ion Battery Anodes,” Electrochimica Acta 284 (2018): 436–443, 10.1016/j.electacta.2018.07.162.

[13] Z. Zhu , S. Wang , J. Du , et al., “Ultrasmall Sn Nanoparticles Embedded in Nitrogen‐Doped Porous Carbon As High‐Performance Anode for Lithium‐Ion Batteries,” Nano Letters 14 (2014): 153–157, 10.1021/nl403631h. 24328829

[14] H. Wu , J. Zhu , L. Liu , et al., “Intercalation and Delamination of Ti_2_SnC with High Lithium Ion Storage Capacity,” Nanoscale 13 (2021): 7355–7361, 10.1039/D0NR06260J. 33889873

[15] J. Zhang , R. Jia , K. B. Tan , et al., “A Review of MAX Series Materials: From Diversity, Synthesis, Prediction, Properties Oriented to Functions,” Nano‐Micro Letters 17 (2025): 173, 10.1007/s40820-025-01673-9. 40025215 PMC11872869

[16] M. Dahlqvist , M. W. Barsoum , and J. Rosen , “MAX Phases—Past, Present, and Future,” Materials Today 72 (2024): 1–24, 10.1016/j.mattod.2023.11.010.

[17] C. Du , X. Chen , W. Zhu , et al., “3D porous SnO_2_/MXene as a Superior Anode Material for Li‐ion and Na‐ion Battery,” Journal of Electroanalytical Chemistry 967 (2024): 118481, 10.1016/j.jelechem.2024.118481.

[18] S. Jolly , S. Husmann , V. Presser , and M. Naguib , “Growth of titania and tin oxide From Ti_2_SnC via rapid thermal oxidation in air for lithium‐ion battery Application,” Journal of the American Ceramic Society 106 (2023): 3261–3271, 10.1111/jace.19010.

[19] S. Zhao , Y. Dall'Agnese , X. Chu , X. Zhao , Y. Gogotsi , and Y. Gao , “Electrochemical Interaction of Sn‐Containing MAX Phase (Nb_2_SnC) with Li‐Ions,” ACS Energy Letters 4 (2019): 2452–2457, 10.1021/acsenergylett.9b01580.

[20] Y. Li , G. Ma , H. Shao , et al., “Electrochemical Lithium Storage Performance of Molten Salt Derived V_2_SnC MAX Phase,” Nano‐Micro Letters 13 (2021): 158, 10.1007/s40820-021-00684-6. 34292406 PMC8298715

[21] J. Tang , W. Zheng , H. Zhang , et al., “Ti_2_SnC MAX Phase with In‐situ Generated Sn Nanoparticles for Lithium‐ion Storage,” Chemical Engineering Journal 503 (2025): 158487, 10.1016/j.cej.2024.158487.

[22] X. Chen , Y. Zhu , X. Zhu , et al., “Partially Etched Ti_3_AlC_2_ as a Promising High‐Capacity Lithium‐Ion Battery Anode,” Chemsuschem 11 (2018): 2677–2680, 10.1002/cssc.201801200. 29943423

[23] Y. Li , J. Lu , M. Li , et al., “Multielemental Single–atom‐thick A Layers in Nanolaminated V_2_(Sn,A)C (A=Fe, Co, Ni, Mn) for Tailoring Magnetic Properties,” Proceedings of the National Academy of Sciences 117 (2020): 820–825, 10.1073/pnas.1916256117. PMC6969549 31879341

[24] C. Hu , H. Dong , Y. Li , et al., “Self‐Reconstruction of Single‐Atom‐Thick A Layers in Nanolaminated MAX Phases for Enhanced Oxygen Evolution,” Advanced Functional Materials 33 (2023): 2211530, 10.1002/adfm.202211530.

[25] B. Gou , L. Wang , B. Ye , et al., “Low‐temperature Synthesis of Pure‐phase Ti_3_(Al, Fe)C_2_ Solid Solution with Magnetic Monoatomic Layers by Replacement Reaction,” Journal of Materials Science: Materials in Electronics 32 (2021): 13081–13088, 10.1007/s10854-021-05761-5.

[26] S. Zhu , Y. Li , M. Yang , et al., “Extraordinary Structural Reconstruction of Nanolaminated Ta_2_FeC MAX Phase for Enhanced Oxygen Evolution Performance,” Small 20 (2024): 2401022, 10.1002/smll.202401022. 38809081

[27] T. Lapauw , B. Tunca , T. Cabioc'h , J. Vleugels , and K. Lambrinou , “Reactive Spark Plasma Sintering of Ti_3_SnC_2_, Zr_3_SnC_2_ and Hf_3_SnC_2_ using Fe, Co or Ni Additives,” Journal of the European Ceramic Society 37 (2017): 4539–4545, 10.1016/j.jeurceramsoc.2017.06.041.

[28] N. Ouabadi , V. Gauthier‐Brunet , T. Cabioc'h , G.‐P. Bei , and S. Dubois , “Formation Mechanisms of Ti_3_SnC_2_ Nanolaminate Carbide Using Fe as Additive,” Journal of the American Ceramic Society 96 (2013): 3239–3242, 10.1111/jace.12427.

[29] M. Fattahi and M. Zarezadeh Mehrizi , “Formation mechanism for synthesis of Ti_3_SnC_2_ MAX phase,” Materials Today Communications 25 (2020): 101623, 10.1016/j.mtcomm.2020.101623.

[30] J. Ding , P. Zhang , W. B. Tian , et al., “The Effects of Sn Content on the Microstructure and the Formation Mechanism of Ti_2_SnC Powder by Pressureless Synthesis,” Journal of Alloys and Compounds 695 (2017): 2850–2856, 10.1016/j.jallcom.2016.11.398.

[31] C. Lu , Y. Wang , X. Wang , and J. Zhang , “Synthesis of Ti_2_SnC Under Optimized Experimental Parameters of Pressureless Spark Plasma Sintering Assisted by Al Addition,” Advances in Materials Science and Engineering 2018 (2018): 9861894, 10.1155/2018/9861894.

[32] B. Yao , S. Li , W. Zhang , et al., “Self‐healing Behavior of Ti_2_AlC at a Low Oxygen Partial Pressure,” Journal of Advanced Ceramics 11 (2022): 1687–1695, 10.1007/s40145-022-0640-0.

[33] S. Zhu , Y. Li , D. Liu , Q. Huang , and Y. Kuang , “Excellent CoO_x_H_y_/C Oxygen Evolution Catalysts Evolved From the Rapid In Situ Electrochemical Reconstruction of Cobalt Transition Metals Doped Into the V_2_SnC MAX Phase at A Layers,” ACS Applied Energy Materials 6 (2023): 1116–1125, 10.1021/acsaem.2c03810.

[34] I. Ostroman , N. Vallana , S. Marchionna , et al., “Oxidized Ti_3_Al_(1‐x)_SnxC_2_ MAX Phases as Negative Electrode Materials for Sodium Ion Batteries,” Journal of Power Sources 624 (2024): 235543, 10.1016/j.jpowsour.2024.235543.

[35] Z. Tian , P. Zhang , W. Sun , B. Yan , and Z. Sun , “Vegard's Law Deviating Ti_2_(Sn_x_Al_1− x_)C Solid Solution with Enhanced Properties,” Journal of Advanced Ceramics 12 (2023): 1655–1669, 10.26599/JAC.2023.9220779.

[36] I. Ostroman , C. Ferrara , S. Marchionna , et al., “Highly Reversible Ti/Sn Oxide Nanocomposite Electrodes for Lithium Ion Batteries Obtained by Oxidation of Ti_3_Al_(1‐x)_Sn_x_C_2_ Phases,” Small Methods 7 (2023): 2300503, 10.1002/smtd.202300503. 37452230

[37] J. Ji , X. Zhan , X. Jiang , et al., “Synthesis and DFT Calculations of High Entropy Six‐component Ti‐based MAX of (Ti, M)_2_AlC (M=Zr, Nb, Ta, Mo, W),” Vacuum 239 (2025): 114332, 10.1016/j.vacuum.2025.114332.

[38] A. L. Patterson , “X‐Ray Diffraction Procedures for Polycrystalline and Amorphous Materials,” Journal of the American Chemical Society 77 (1955): 2030–2031, 10.1021/ja01612a110.

[39] M. W. Barsoum , “The MN+1AXN phases: A new class of solids,” Progress in Solid State Chemistry 28 (2000): 201–281, 10.1016/S0079-6786(00)00006-6.

[40] Y. Xie , G. Chen , Y. Tang , et al., “Unraveling the Ionic Storage Mechanism of Flexible Nitrogen‐Doped MXene Films for High‐Performance Aqueous Hybrid Supercapacitors,” Small 20 (2024): 2405817, 10.1002/smll.202405817. 39377313

[41] H. Wei , L. Chen , H. Ding , Y. Li , Z. Chai , and Q. Huang , “Dual‐Phase Structure Through Selective Etching of the Double A‐Element MAX Phase in Lewis Acidic Molten Salts,” The Journal of Physical Chemistry Letters 15 (2024): 4486–4493, 10.1021/acs.jpclett.4c00785. 38634523

[42] M. A. Hadi , S. R. G. Christopoulos , A. Chroneos , S. H. Naqib , and A. K. M. A. Islam , “DFT Insights into the Electronic Structure, Mechanical Behaviour, Lattice dynamics and Defect Processes in the First Sc‐based MAX phase Sc_2_SnC,” Scientific Reports 12 (2022): 14037, 10.1038/s41598-022-18336-z. 35982080 PMC9388654

[43] S. Zhao , H. Xiao , Y. Li , et al., “Multi‐stage Phase Transformation Pathways in MAX Phases,” Nature Communications 16 (2025): 1554, 10.1038/s41467-025-56921-8. PMC11814106 39934161

[44] M. Xiao , W. Li , M. Yu , et al., “Enhanced Electronic Conductivity and Ionic Conductivity of Li_2_S by Doping Strategy,” Matter 8 (2025): 101934, 10.1016/j.matt.2024.11.028.

[45] H. Adenusi , G. A. Chass , S. Passerini , K. V. Tian , and G. Chen , “Lithium Batteries and the Solid Electrolyte Interphase (SEI)—Progress and Outlook,” Advanced Energy Materials 13 (2023): 2203307, 10.1002/aenm.202203307.

[46] L. Sun , H. Si , Y. Zhang , et al., “Sn‐SnO_2_ Hybrid Nanoclusters Embedded in Carbon Nanotubes with Enhanced Electrochemical Performance for Advanced Lithium Ion Batteries,” Journal of Power Sources 415 (2019): 126–135, 10.1016/j.jpowsour.2019.01.063.

[47] X. Xu , D. Sha , Z. Tian , et al., “Lithium Storage Performance and Mechanism of Nano‐sized Ti_2_InC MAX phase,” Nanoscale Horizons 8 (2023): 331–337, 10.1039/D2NH00489E. 36621903

[48] J. Luo , X. Tao , J. Zhang , et al., “Sn 4+ Ion Decorated Highly Conductive Ti_3_C_2_ MXene: Promising Lithium‐Ion Anodes with Enhanced Volumetric Capacity and Cyclic Performance,” ACS Nano 10 (2016): 2491–2499, 10.1021/acsnano.5b07333. 26836262

[49] X. Xuan , Y. Xie , Y. Tang , et al., “Revealing the Ionic Storage Mechanisms of Mo_2_VC_2_T_z_ (MXene) in Multiple Aqueous Electrolytes for High‐performance Supercapacitors,” Chemical Engineering Journal 519 (2025): 165537, 10.1016/j.cej.2025.165537.

[50] J. Y. Huang , L. Zhong , C. M. Wang , et al., “In Situ Observation of the Electrochemical Lithiation of a Single SnO_2_ Nanowire Electrode,” Science 330 (2010): 1515–1520, 10.1126/science.1195628. 21148385

[51] X. Liu , Y. Liu , Z. Shao , et al., “Dynamic Gradient Oxygen Layer Enables Stable Sn Anode for Lithium Storage,” Advanced Materials 37 (2025): 2505136, 10.1002/adma.202505136. 40509654

[52] J. Wang , J. Polleux , J. Lim , and B. Dunn , “Pseudocapacitive Contributions to Electrochemical Energy Storage in TiO_2_ (Anatase) Nanoparticles,” The Journal of Physical Chemistry C 111 (2007): 14925–14931, 10.1021/jp074464w.

