# Supplementary material for: Highly Robust Sn‐Based MAX Anodes Constructed via Ti6C Octahedral Outer Immobilization and A‐Layer Fe Inner Anchoring for Long‐Life Lithium‐Ion Batteries
Source: Adv Sci (Weinh). 2026 Apr 10;13(34):e75085. doi: 10.1002/advs.75085 (PMC13285164; doi:10.1002/advs.75085)
Supplement: Supplementary file 1 — Supporting File: advs75085‐sup‐0001‐SuppMat.docx. [file ADVS-13-e75085-s001.docx]

**Supporting Information**

**Highly Robust Sn-Based MAX Anodes Constructed *via* Ti_6_C Octahedral Outer Immobilization and A-Layer Fe Inner Anchoring for Long-Life Lithium-Ion Batteries**

*Yu-ang Lei, Yuchen Guo, Linkai He, Yi Tang*, Junhui Zou, Yangyang Xie,* *Xiaodie Xuan, Zhao Bi, Yu Wang, Ting Liu, Yunqing Kang,* *Taotao Ai*, Yusuke Yamauchi*, Chenhui Yang**

Y. Lei, Y. Guo, L. He, J. Zou, Y. Xie, X. Xuan, Z. Bi, Y. Wang, Prof. C. Yang

School of Chemistry and Chemical Engineering, Northwestern Polytechnical University, No. 1 Dongxiang Road, Chang’an District, Xi’an, Shaanxi 710129, P.R. China

Prof. Y. Tang

College of Materials Science and Engineering, Xi’an University of Science and Technology, Xi’an, Shaanxi 710054, P. R. China

Ting Liu

State Key Laboratory of Solidification Processing, Center for Nano Energy Materials, School of Materials Science and Engineering, Northwestern Polytechnical University, No. 127 Youyi West Road, Beilin District, Xi’an, Shaanxi 710072, P.R. China

Prof. T. Ai

School of Materials Science and Engineering, Shaanxi University of Technology, Hanzhong, Shaanxi 723001, P. R. China

Dr. Y. Kang, Prof. Y. Yamauchi

Department of Materials Process Engineering, Graduate School of Engineering, Nagoya University, Nagoya 464–8603, Japan

Prof. Y. Yamauchi

Australian Institute for Bioengineering and Nanotechnology (AIBN), The University of Queensland, Brisbane, Queensland, 4072 Australia

***Corresponding Authors.**

Email:

yangch@nwpu.edu.cn, ORCID: 0000-0001-5563-2695 (C. Yang);

y.yamauchi@nagoya-u.jp, ORCID: 0000-0001-7854-927X (Y. Yamauchi);

aitaotao0116@126.com, ORCID: 0000-0002-5795-5751 (T. Ai);

tangyi150@xust.edu.cn, ORCID: 0000-0002-1511-2422 (Y. Tang)

**Table of contents**

[**1. Experimental procedures** 3](#_Toc225969782)

[**1.1. Synthesis of Ti_2_Sn_1-_*_x_*Fe*_x_*C MAX powders** 3](#_Toc225969783)

[**1.2. Materials characterizations** 3](#_Toc225969784)

[**1.3. Electrochemical measurements** 3](#_Toc225969785)

[**2. Theoretical calculations** 5](#_Toc225969786)

[**3. Electrochemical performance calculation** 6](#_Toc225969787)

[**4. Figures** 8](#_Toc225969788)

[**5. Tables** 17](#_Toc225969789)

[**References** 19](#_Toc225969790)

**1. Experimental procedures**

**1.1. Synthesis of Ti_2_Sn_1-_*_x_*Fe*_x_*C MAX powders**

The Ti_2_Sn_1-_*_x_*Fe*_x_*C (0≤x≤0.33) powders were prepared by Ar atmosphere sintering of commercial Ti (99.99 %, ≤48 μm, Aladdin), Sn (99.5 %, ~74 μm, Sinopharm), Fe (99.9 %, ~10 μm, 3A), and TiC (99 %, 2–4 μm, Aladdin) powders with a Ti/Sn/Fe/TiC molar ratio of 1 :(1-*x*) :*x* :1. The mixture was ground several times in an agate mortar and pestle. The mixture was then annealed at 1200 °C for 2 h under argon gas with a heating rate of 5 °C min^-1^. Residual metals were removed from the prepared powders with 3 M HCl solution, and the powders were filtered and dried at 50 °C. The powder was then ball-milled for 300 min at 550 rpm using an agate jar and grinding balls in a 10:1 ball-to-material ratio. The Ti_2_Sn_1-_*_x_*Fe*_x_*C powders were thus obtained for further study.

**1.2. Materials characterizations**

The X-ray diffraction (XRD) patterns were recorded using a D8 ADVANCE A25 X-ray diffractometer (Bruker AXS GmbH, Germany) with Cu Kα radiation in the 2θ range of 5–80°. X-ray photoelectron spectroscopy (XPS) measurements were performed using a Thermo Fisher ESCALAB Xi+ spectrometer with an Al Kα excitation source. Morphological and elemental profiles of the samples were recorded using field-emission scanning electron microscopy (SEM, FEI Ltd, Verios G4) with energy-dispersive X-ray analysis (EDAX) and transmission electron microscopy (TEM, FEI F200X). Magnetic properties were measured using a vibrating-sample magnetometer (VSM, Lakeshore 8604, Quantum Design, USA) in a magnetic field ranging from −20 kOe to +20 kOe.

**1.3. Electrochemical measurements**

Standard CR2032 coin cells (Canrd) were assembled in an argon-filled glove box with H₂O and O₂ concentrations maintained below 0.01 ppm to avoid moisture contamination. The working electrodes were prepared by mixing the active material, polyvinylidene fluoride (PVDF), and carbon black in a mass ratio of 8:1:1 using 1-methyl-2-pyrrolidinone (NMP) as the solvent. The resulting slurry was coated onto copper foil (20 μm, Lizhiyuan Trade), followed by vacuum drying at 110 °C for 12 h to remove the NMP. The loading of active material on the copper foil was approximately 0.8-1.0 mg cm^-2^. Half-cells were assembled using Li foil as counter and reference electrodes and a polyethylene separator. The electrolyte used was 1 M LiPF_6_ dissolved in a mixture of ethylene carbonate (EC) and dimethyl carbonate (DMC) (1:1 v/v, Mojiesi Trade). Galvanostatic charge/discharge measurements were conducted on a LANHE CT3002A tester (LAND, China) within a voltage range of 0.01 to 3.00 V (vs. Li⁺/Li). Cyclic voltammetry (CV) was performed on a CORRTEST CS310X electrochemical workstation. CV curves were recorded at scanning rates of 0.1-5.0 mV s^-1^. Electrochemical impedance spectroscopy (EIS) was performed on a CHI660E electrochemical workstation. EIS was carried out at open circuit potential, with an amplitude at 5 mV, at a frequency range from 100 kHz to 10 mHz.

**2. Theoretical calculations**

Density functional theory (DFT) calculations were performed using the Vienna Ab initio Simulation Package (VASP, version 6.4.3)^[1]^ with the projector augmented wave (PAW)^[2]^ method and plane-wave basis sets^[3]^. A plane-wave kinetic energy cutoff of 750 eV was employed throughout all calculations. The Brillouin zone was sampled using Monkhorst-Pack scheme with a k-point mesh of 5x5x1 in the Gamma-centered grids for the structural relaxations^[4]^. The structures were fully relaxed until the maximum force was less than 10^-4^ eV and the energy convergence criterion was 10^-5^ eV. Crystal orbital Hamilton populations analyses were performed using the Local-Orbital Basis Suite Towards Electronic-Structure Reconstruction (LOBSTER, version 5.1.1) with pbeVaspFit2015 basis sets^[5]^.

**3. Electrochemical performance calculation**

To further investigate the contribution of the pseudocapacitance to the electrochemical properties, we have carried out CV tests on Ti_2_Sn_1-_*_x_*Fe*_x_*C at different scan rates. It can be assumed that the current response is exponentially related to the scan rate as follows^[6-7]^:

| ***i* = a*v^b^*** | (S1) |
| --- | --- |
| **Log*i* = Loga + *b**Log*v*** | (S2) |

Here, a is a constant, *v* is the scan rate, and *b* is a value ranging from 0.5 (indicating a diffusion-controlled contribution) to 1 (indicating a capacitive contribution). A *b* value of 0.5 suggests that the current is controlled by semi-infinite linear diffusion, while a *b* value of 1 suggests that the current is controlled by the surface.

In order to further investigate Li-ion storage mechanism of the Ti_2_Sn_1-_*_x_*Fe*_x_*C, the contributing behavior of the surface capacitance was explored using the following equation^[7]^:

| ***i* = *k*_1_*v + k*_2_*v*^1/2^** | (S3) |
| --- | --- |

Where k_1_ and k_2_ are constants and k_1_v and k_2_v^1/2^ correspond to capacitance-controlled and diffusion-controlled processes, respectively.

The calculation of the ion diffusion coefficient based on the galvanostatic intermittent titration technique (GITT) uses the following equation^[6]^:

| ***D* *^GITT^* =(4 /πτ)(*m_B_ V_M_* / *m_B_ S*)^2^ (Δ*E_s_*/Δ*E_τ_*)^2^** | (S4) |
| --- | --- |

Where, *τ* is the relaxation time (also known as pulse time), *n* is the molarity of the active substance, *V_m_* is the molar volume, and A is the contact area (cm^-2^) of the electrode/electrolyte, *∆E_s_* is the voltage change due to the pulse (V); *∆E_τ_* is the voltage change due to the constant current charge (discharge) (V), *L* is the diffusion length (cm).

**4. Figures**


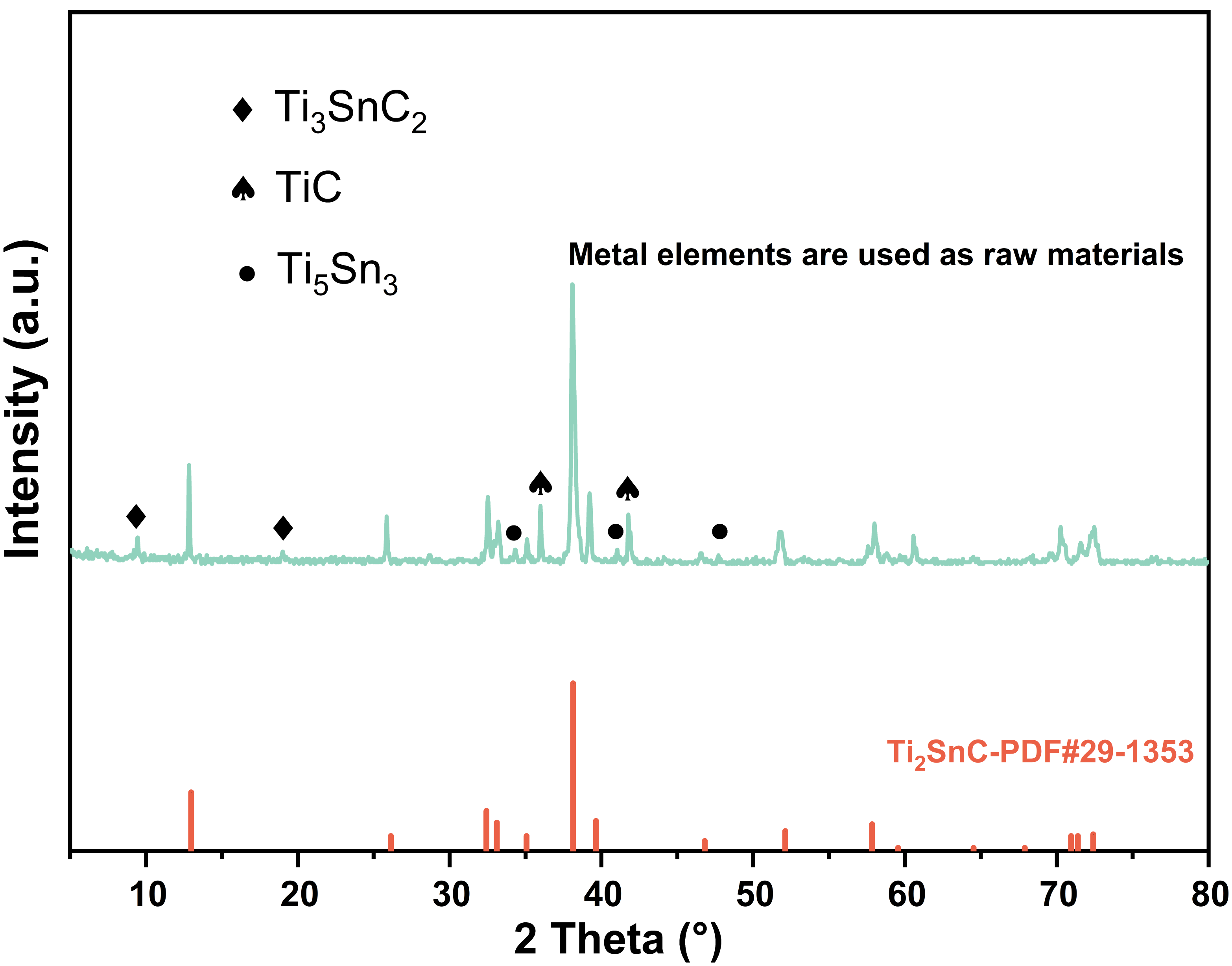


**Figure S1.** XRD pattern of the sample prepared by firing using Ti, Sn, Fe, C as raw materials.


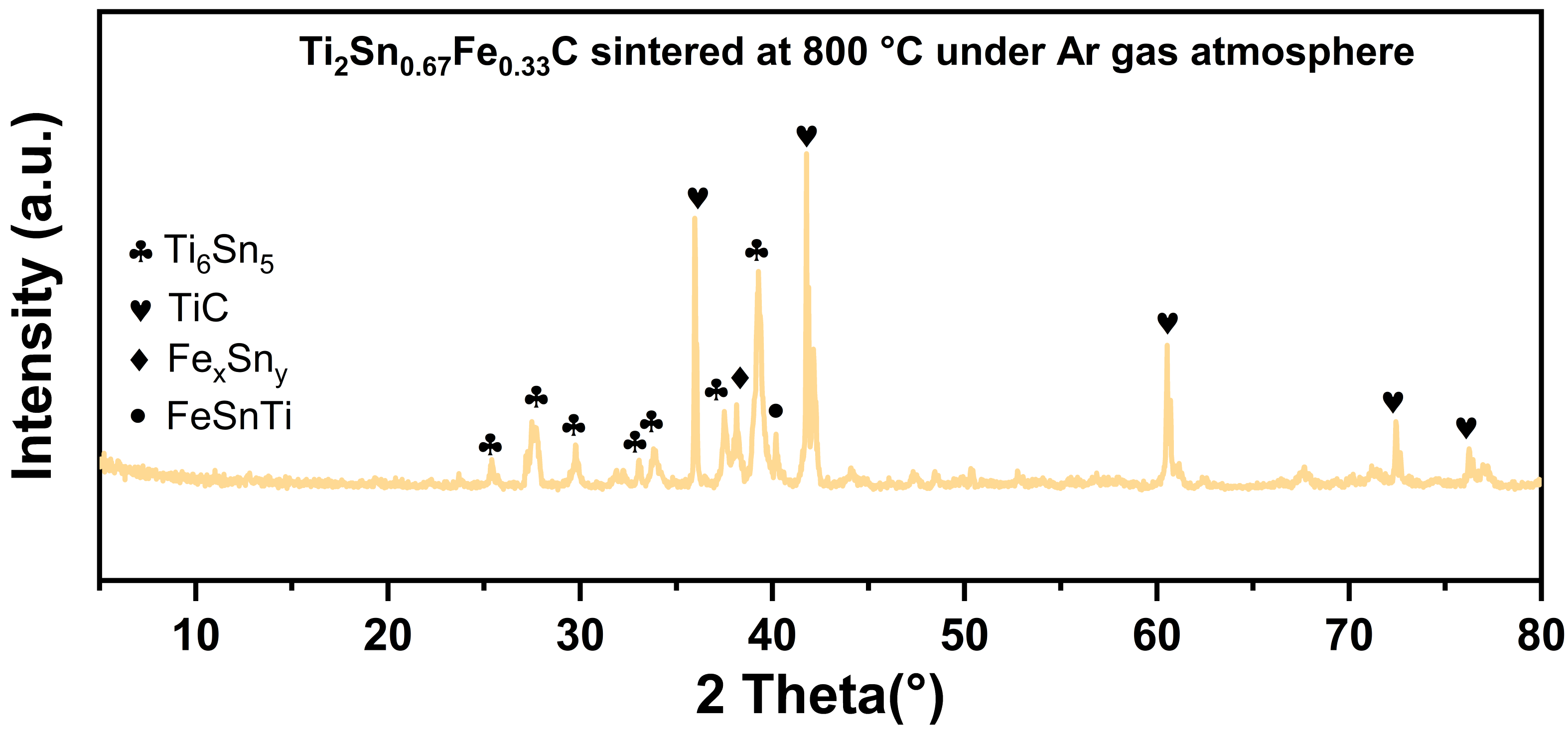


**Figure S2.** XRD pattern of Ti_2_Sn_0.67_Fe_0.33_C at the intermediate stage of sintering (800°C).

**
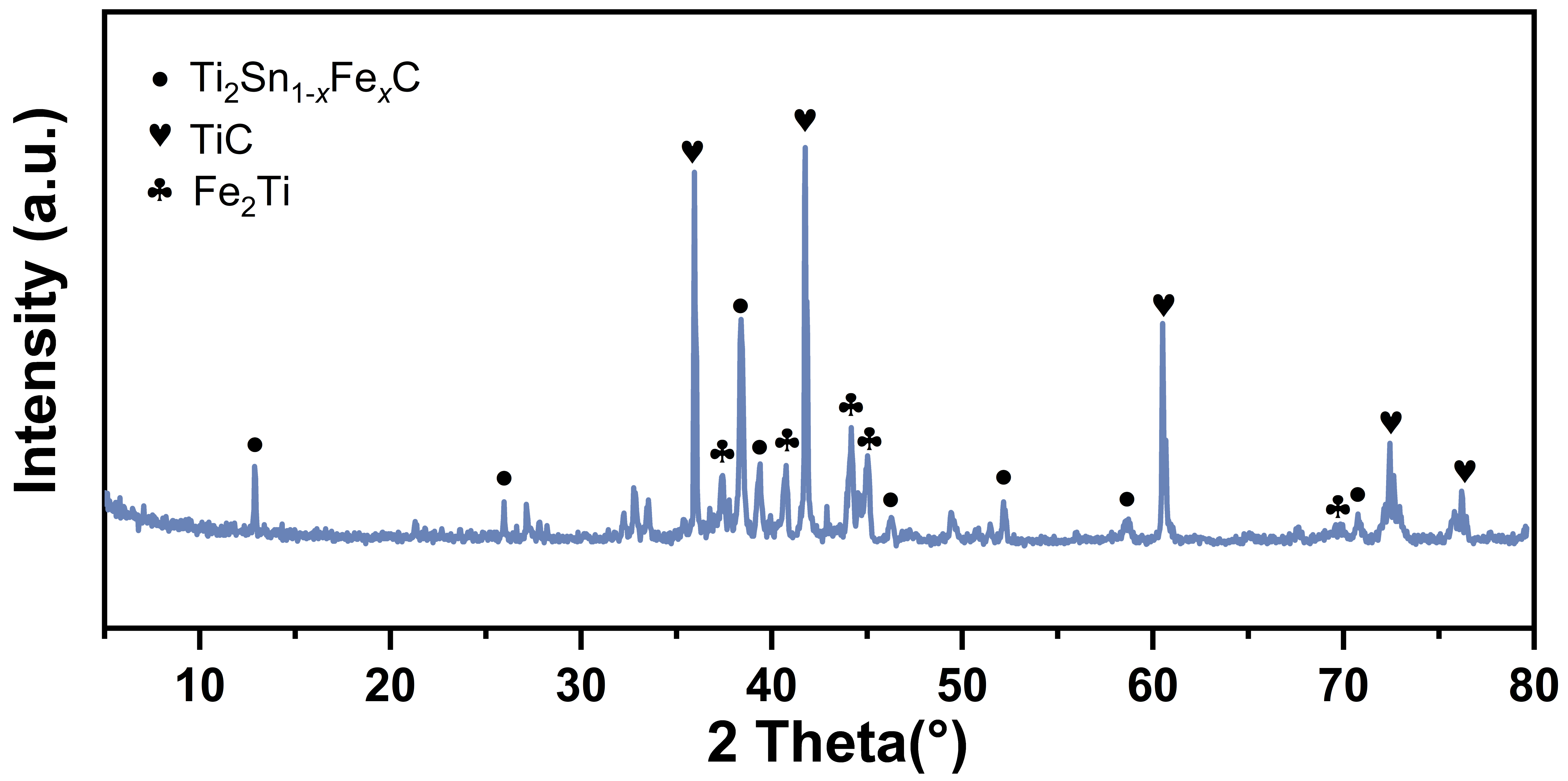
**

**Figure S3.** XRD pattern of the sample with a raw material ratio of TiC: Ti: Sn: Fe = 1 :1 :0.5 :0.5.


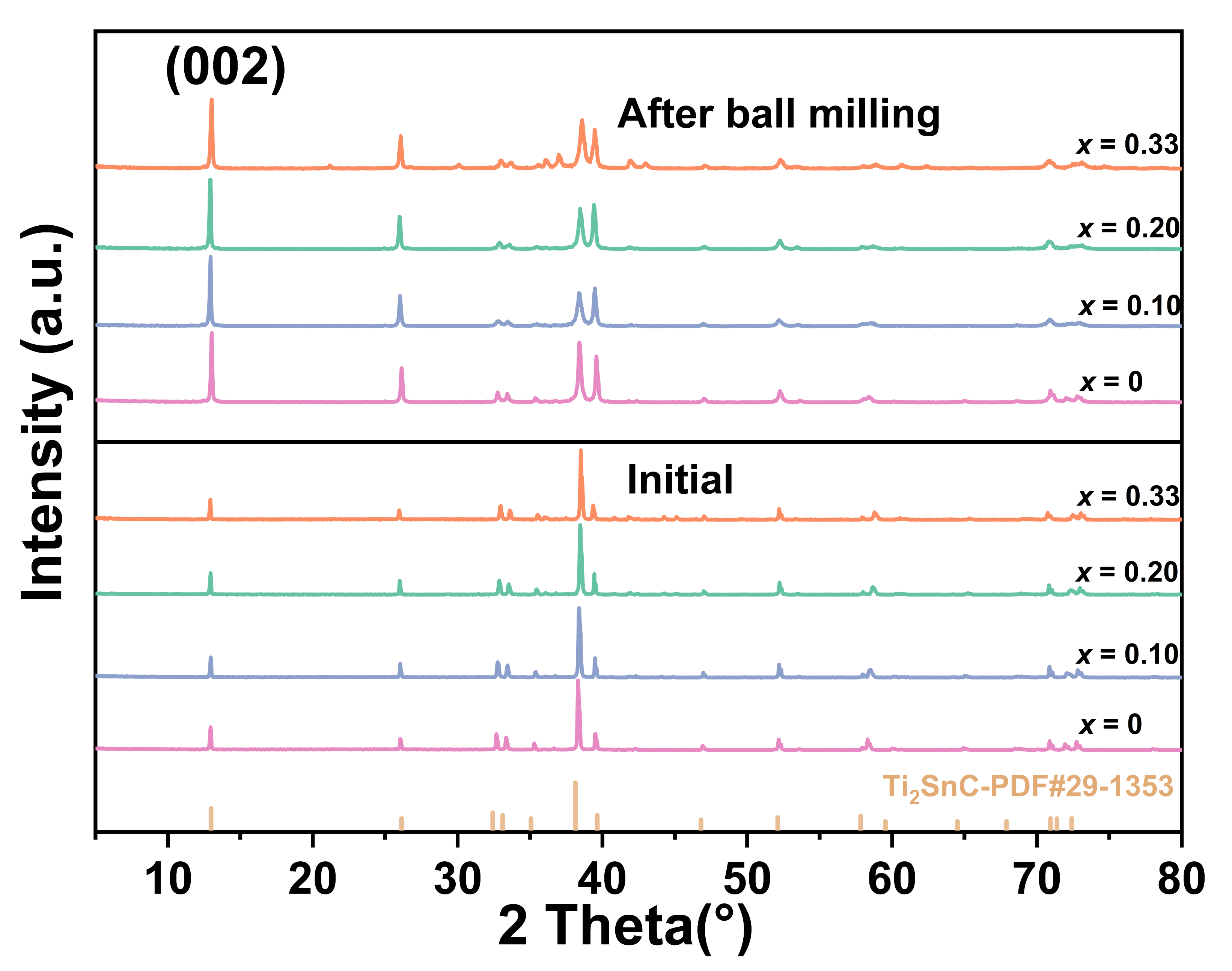


**Figure S4.** XRD patterns of different Ti_2_Sn_1-_*_x_*Fe*_x_*C phases before and after ball milling.


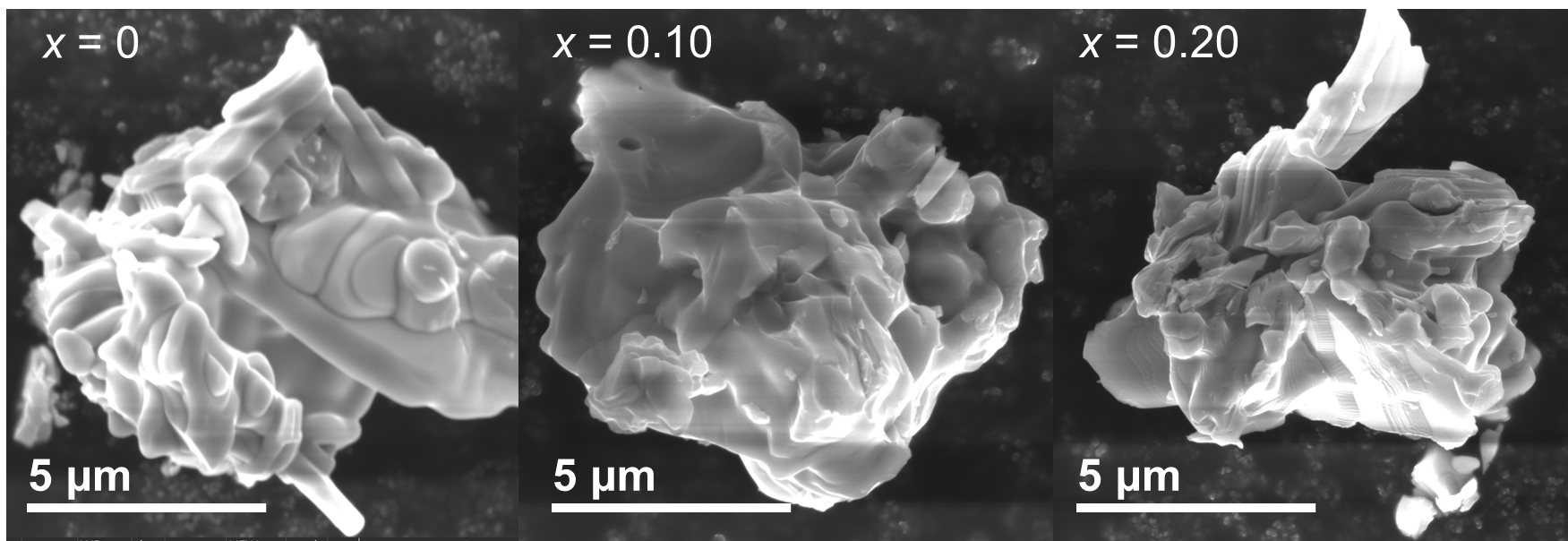


**Figure S5.** SEM images of the solid-solution Ti_2_Sn_1-_*_x_*Fe*_x_*C (*x* = 0, 0.10, 0.20).


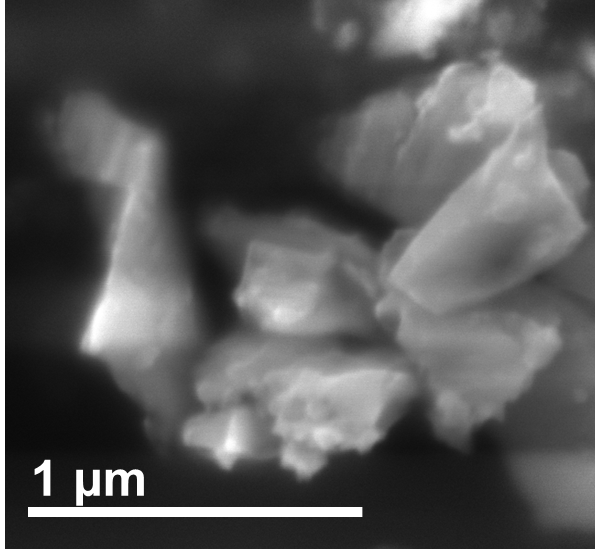


**Figure S6.** SEM image of Ti_2_Sn_0.67_Fe_0.33_C after ball milling.


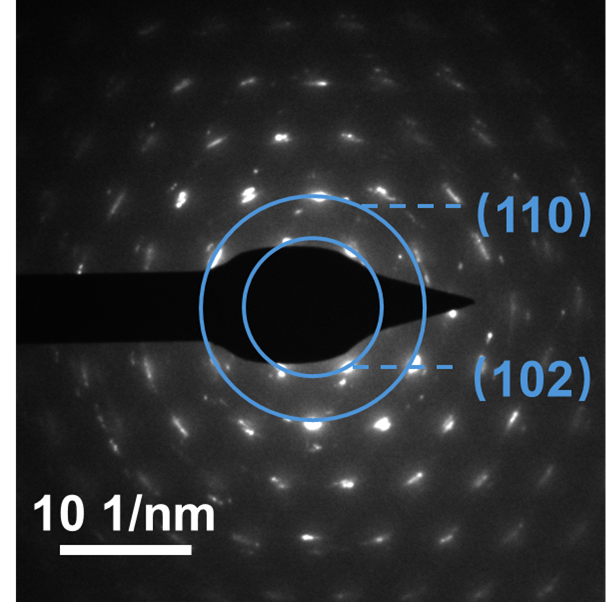


**Figure S7.** Selected area electron diffraction (SAED) image of Ti_2_Sn_0.67_Fe_0.33_C.


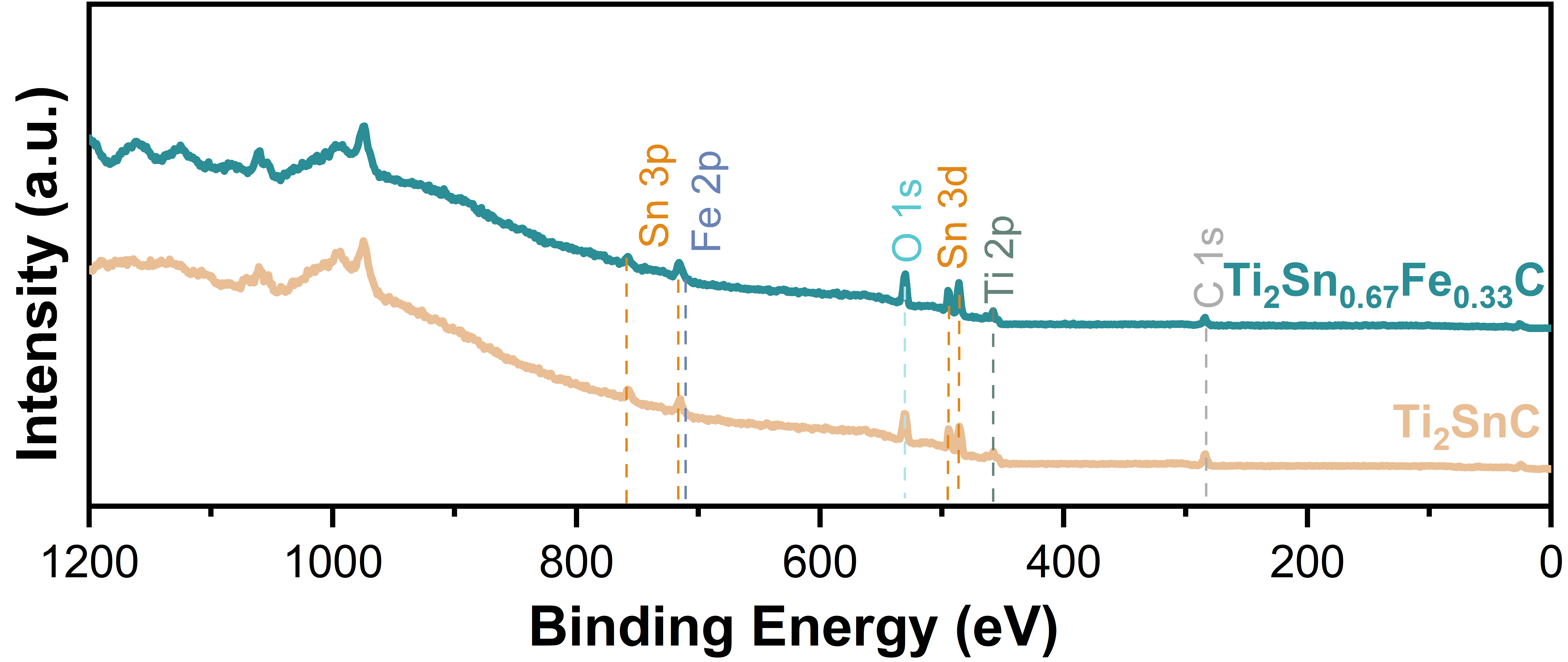


**Figure S8.** Full XPS spectra of Ti_2_SnC and Ti_2_Sn_0.67_Fe_0.33_C.


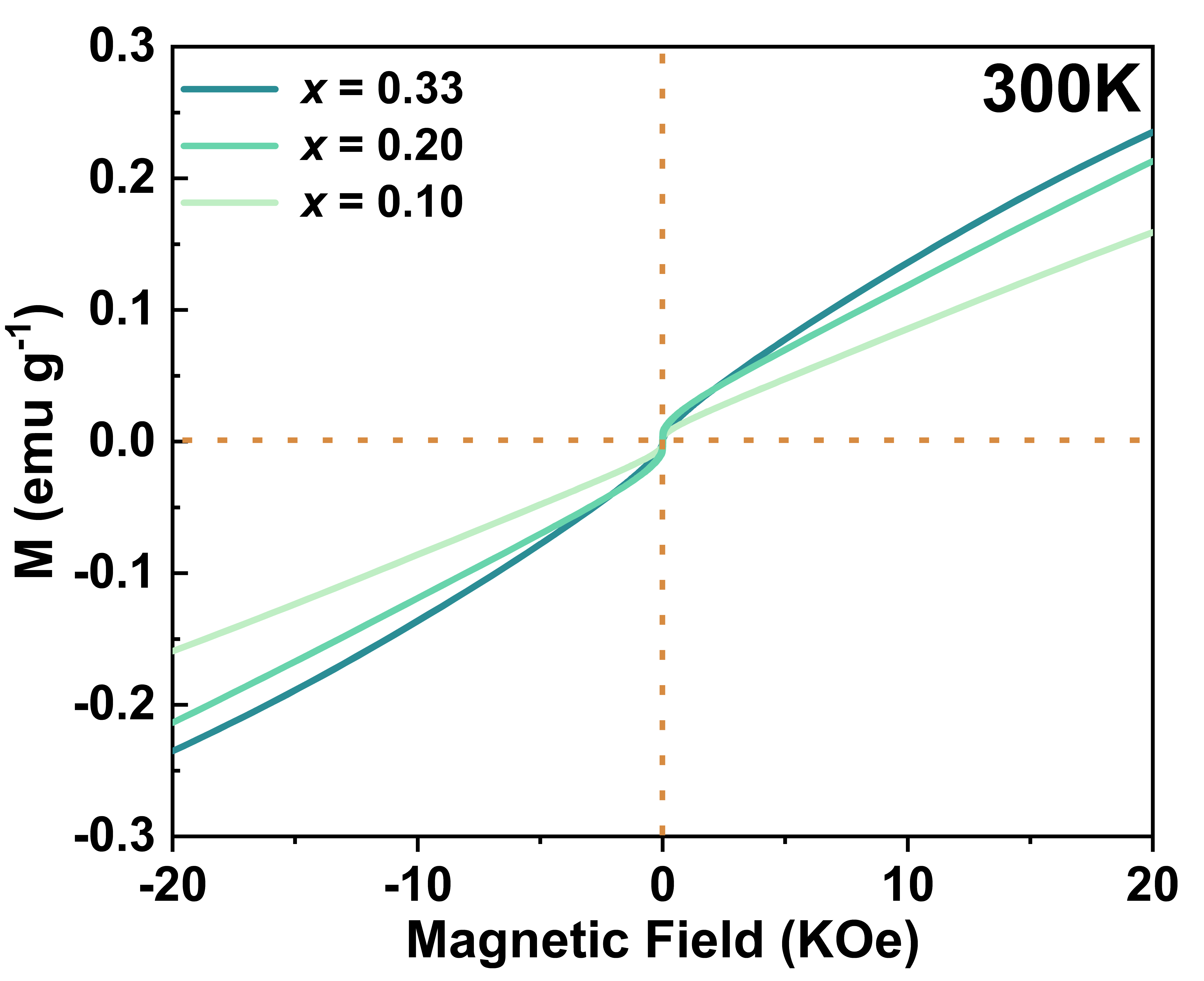


**Figure S9.** Magnetic hysteresis loops of Ti_2_Sn_1-_*_x_*Fe*_x_*C in the range from −10 kOe to 10 kOe.

.



**Figure S10.** The rate performance of four Ti_2_Sn_1-_*_x_*Fe*_x_*C electrodes before cycling at different current densities.





**Figure S11.** Nyquist plots of Ti_2_Sn_0.8_Fe_0.2_C and Ti_2_Sn_0.9_Fe_0.1_C electrodes.





**Figure S12.** CV curves between 0.01 and 3 V of four Ti_2_Sn_1-_*_x_*Fe*_x_*C electrodes before cycling at 0.1 mV s^-1^.

**

**

**Figure S13.** CV curves of Ti_2_Sn_0.67_Fe_0.33_C at 0.1 mV s^-1^ between 0.01 and 3.00 V.

The XPS Ti 2p spectrum reveals the presence of Ti^4+^ arising from partial oxidation of the MAX surface during ball milling, while the coexisting Ti^2+^and Ti^3+^species originate from the unoxidized MAX phase bulk.


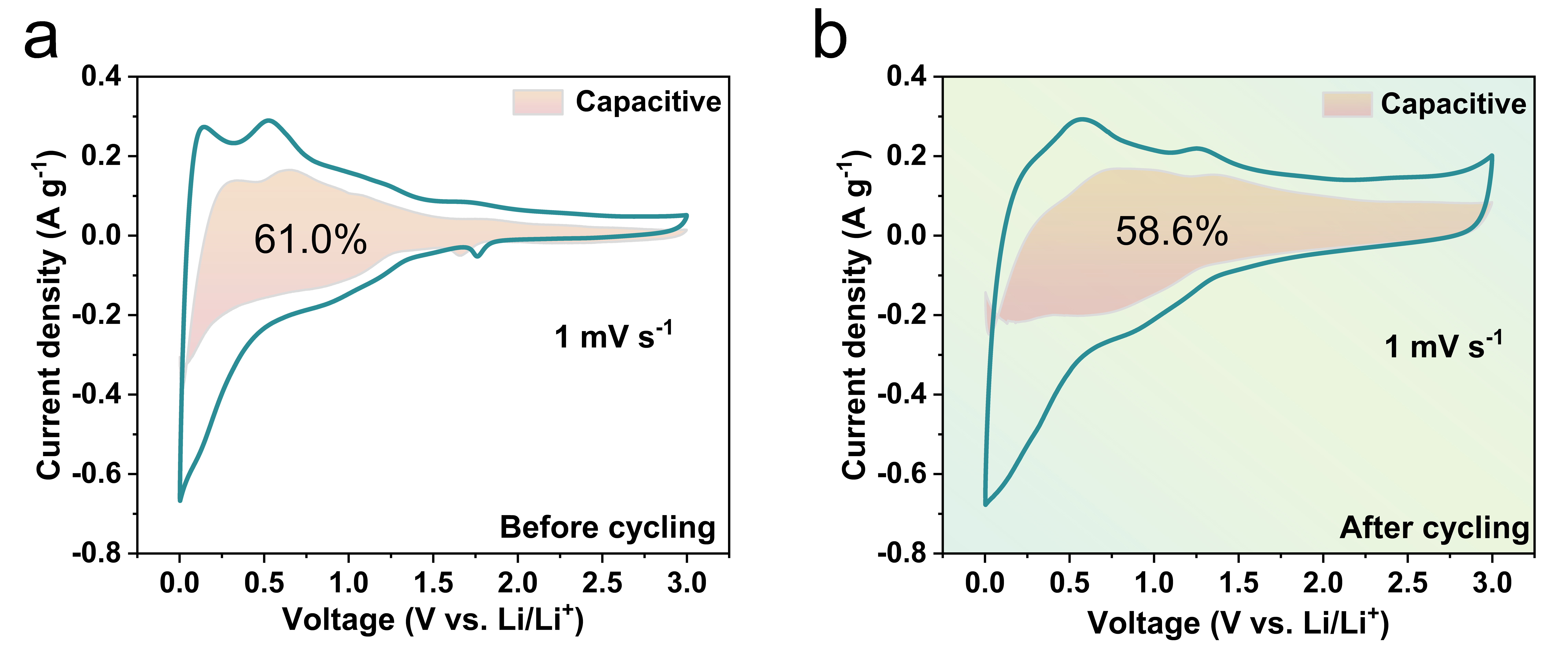


**Figure S14.** The capacitive contribution to charge storage the of Ti_2_Sn_0.67_Fe_0.33_C electrode a) before and b) after 2000 cycles at a scan rate of 1 mV s^−1^.


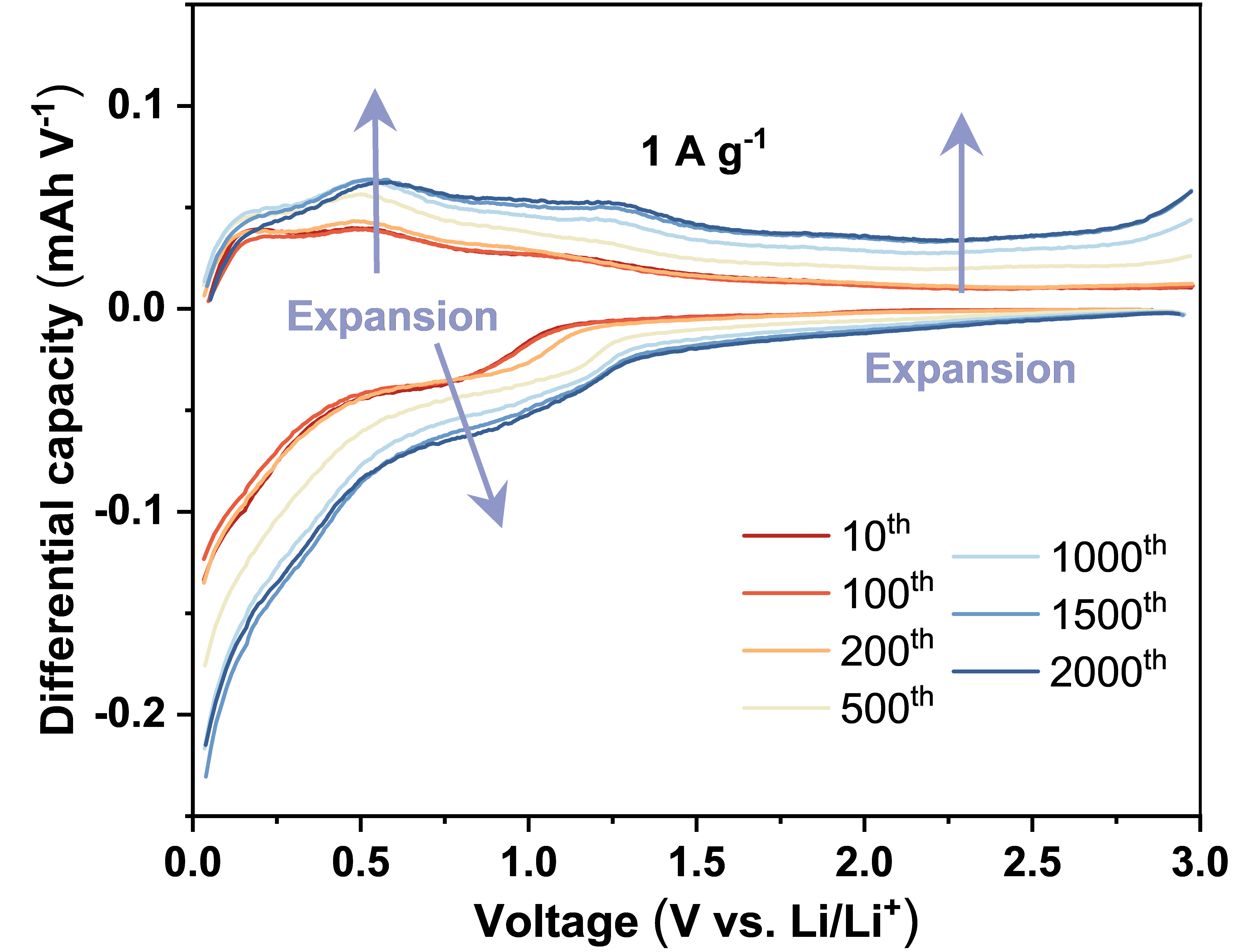


**Figure S15.** dQ/dV profiles of the Ti_2_Sn_0.67_Fe_0.33_C electrode at 1 A g^-1^ under different cycles.


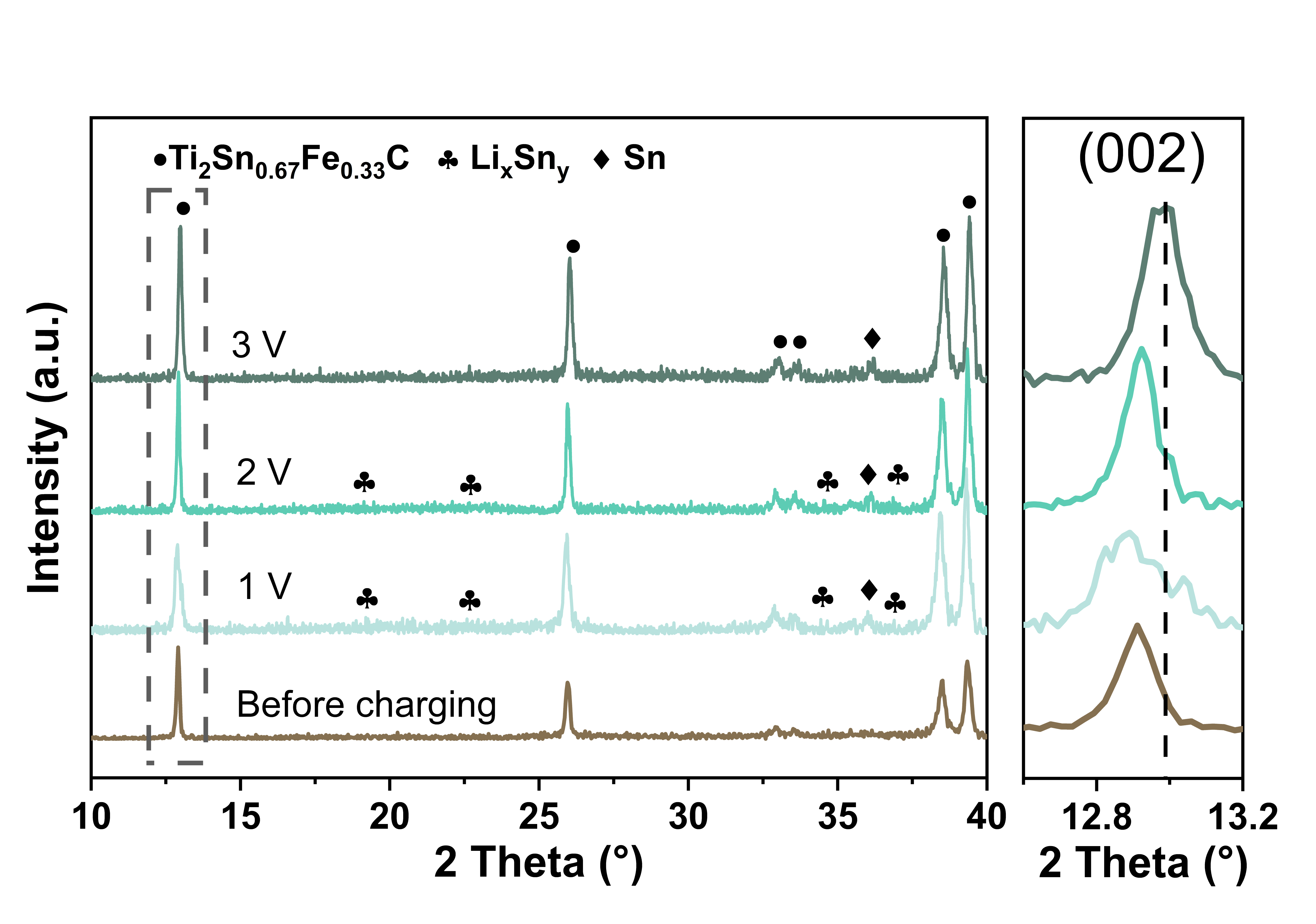


**Figure S16.** *Ex-situ* XRD patterns of the Ti_2_Sn_0.67_Fe_0.33_C electrode charged to different voltages.

**5. Tables**

**Table S1.** Compositional analysis of as-synthesized Ti_2_Sn_1-_*_x_*Fe*_x_*C.

| **Ti_2_Sn_1-_*_x_*Fe*_x_*C samples** | **Ti (at.%)** | **Sn (at.%)** | **Fe (at.%)** | **C (at.%)** | **Actual value** |
| --- | --- | --- | --- | --- | --- |
| *x* = 0 | 50.09 | 27.83 | 0 | 22.09 | 0 |
| *x* = 0.10 | 47.74 | 23.77 | 2.51 | 25.98 | 0.10 |
| *x* = 0.20 | 48.72 | 23.84 | 6.59 | 20.85 | 0.22 |
| *x* = 0.33 | 42.70 | 20.43 | 9.37 | 27.49 | 0.31 |

**Table S2.** Cyclic performance of four Ti_2_Sn_1-_*_x_*Fe*_x_*C electrodes at 1 A g^-1^.

| **Ti_2_Sn_1-_*_x_*Fe*_x_*C samples** | **Specific capacity (mAh g^-1^)** | | | | **Average**  **Coulombic efficiency (%)** |
| --- | --- | --- | --- | --- | --- |
|  | **100****^th^ cycle** | **500^th^ cycle** | **1000^th^ cycle** | **2000^th^ cycle** |  |
| *x* = 0 | 44.1 | 43.2 | 54.1 | / | 99.75 |
| *x* = 0.10 | 53.2 | 53.9 | 61.5 | / | 99.65 |
| *x* = 0.20 | 77.4 | 90.5 | 99.8 | / | 99.87 |
| *x* = 0.33 | 103.9 | 151.0 | 179.6 | 210.6 | 99.55 |

**References**

[1] G. Kresse, J. Furthmüller, *Phys. Rev. B* **1996**, *54*, 11169.

[2] G. Kresse, D. Joubert, *Phys. Rev. B* **1999**, *59*, 1758.

[3] G. Kresse, J. Furthmüller, *Comput. Mater. Sci.* **1996**, *6*, 15.

[4] H. J. Monkhorst, J. D. Pack, *Phys. Rev. B* **1976**, *13*, 5188.

[5] S. Maintz, V. L. Deringer, A. L. Tchougréeff, R. Dronskowski, *J. Comput. Chem.* **2016**, *37*, 1030.

[6] X. Xuan, Y. Xie, Y. Tang, J. Zhou, Z. Bi, J. Zou, Y.-a. Lei, J. Gao, L. Li, A. Zhang, C. Yang, *Chem. Eng. J.* **2025**, *519*, 165537.

[7] J. Zhou, N. Chang, Y. Tang, Y. Xie, X. Xuan, Z. Bi, J. Zou, L. Li, M. Liu, C. Yang, *Chem. Eng. J.* **2025**, *519*, 165391.
